# OPERA models for predicting physicochemical properties and environmental fate endpoints

**DOI:** 10.1186/s13321-018-0263-1

**Published:** 2018-03-08

**Authors:** Kamel Mansouri, Chris M. Grulke, Richard S. Judson, Antony J. Williams

**Affiliations:** 1 National Center for Computational Toxicology, Office of Research and Development, U.S. Environmental Protection Agency, Research Triangle Park, NC 27711 USA; 2 Oak Ridge Institute for Science and Education, 1299 Bethel Valley Road, Oak Ridge, TN 37830 USA; 3 Present Address: ScitoVation LLC, 6 Davis Drive, Research Triangle Park, NC 27709 USA

**Keywords:** OPERA, QSAR/QSPR, Physicochemical properties, Environmental fate, OECD principles, Open data, Open source, Model validation, QMRF

## Abstract

The collection of chemical structure information and associated experimental data for quantitative structure–activity/property relationship (QSAR/QSPR) modeling is facilitated by an increasing number of public databases containing large amounts of useful data. However, the performance of QSAR models highly depends on the quality of the data and modeling methodology used. This study aims to develop robust QSAR/QSPR models for chemical properties of environmental interest that can be used for regulatory purposes. This study primarily uses data from the publicly available PHYSPROP database consisting of a set of 13 common physicochemical and environmental fate properties. These datasets have undergone extensive curation using an automated workflow to select only high-quality data, and the chemical structures were standardized prior to calculation of the molecular descriptors. The modeling procedure was developed based on the five Organization for Economic Cooperation and Development (OECD) principles for QSAR models. A weighted k-nearest neighbor approach was adopted using a minimum number of required descriptors calculated using PaDEL, an open-source software. The genetic algorithms selected only the most pertinent and mechanistically interpretable descriptors (2–15, with an average of 11 descriptors). The sizes of the modeled datasets varied from 150 chemicals for biodegradability half-life to 14,050 chemicals for logP, with an average of 3222 chemicals across all endpoints. The optimal models were built on randomly selected training sets (75%) and validated using fivefold cross-validation (CV) and test sets (25%). The CV Q^2^ of the models varied from 0.72 to 0.95, with an average of 0.86 and an R^2^ test value from 0.71 to 0.96, with an average of 0.82. Modeling and performance details are described in QSAR model reporting format and were validated by the European Commission’s Joint Research Center to be OECD compliant. All models are freely available as an open-source, command-line application called OPEn structure–activity/property Relationship App (OPERA). OPERA models were applied to more than 750,000 chemicals to produce freely available predicted data on the U.S. Environmental Protection Agency’s CompTox Chemistry Dashboard.

**Electronic supplementary material:**

The online version of this article (10.1186/s13321-018-0263-1) contains supplementary material, which is available to authorized users.

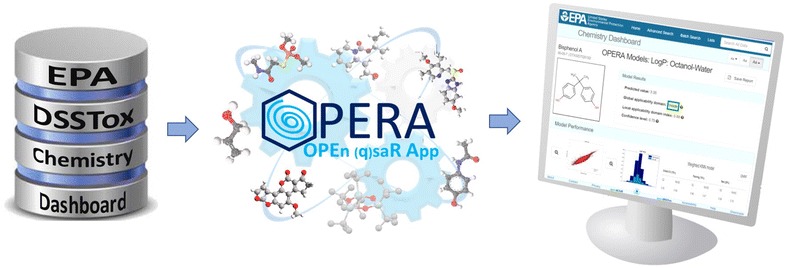

## Background

The increase in the number and quantity of manufactured chemicals finding their way into the environment is proportionally increasing potential exposures of humans and wildlife to potentially harmful substances [1–7]. Due to constraints associated with time, costs, and animal welfare issues, most of these chemicals lack experimentally measured properties [8–11]. To quickly assess large numbers of chemicals for potential toxicity at reasonable cost, the U.S. Environmental Protection Agency (EPA) and other regulatory agencies need to develop new, more efficient testing and evaluation methods [2, 12–18]. Over the past decade, high-throughput screening (HTS) approaches developed by the pharmaceutical industry for drug discovery have been used as alternative approaches to traditional toxicity tests for environmental chemicals [19–22]. At the EPA, since 2007, the National Center for Computational Toxicology (NCCT) has been evaluating HTS approaches through its ToxCast program [9, 22–24]. However, because tens of thousands of chemicals require screening [3, 7, 15, 18, 25], faster and more cost-effective in silico methods such as quantitative structure–activity/property relationships (QSAR/QSPR) modeling approaches [13, 16, 18, 26–28] are needed to prioritize chemicals for testing.

The growing use of QSAR modeling approaches for virtual screening and data gap filling by the scientific community is establishing QSAR models as internationally recognized alternatives to empirical testing by regulatory agencies and organizations such as REACH and the United Nations Globally Harmonized System of Classification and Labeling of Hazardous Chemicals [18, 28–33]. In addition to aiding in prioritization, QSAR models including other calculated descriptors and predicted chemical properties [23, 34] can help overcome difficulties that may arise during in vitro to in vivo extrapolation (IVIVE) or exposure assessment. Therefore, reliable predictions for both physicochemical properties and environmental fate endpoints are needed for risk assessment as well as prioritization for testing, among other applications.

The most widely used chemical properties in toxicological studies, risk assessment, and exposure studies are associated with bioavailability, permeability, absorption, transport, and persistence of chemicals in the body and in the environment [35–39]. These properties (including, but not limited to, the octanol–water partition coefficient, water solubility, melting point, bioconcentration factor, and biodegradability) have been extensively modeled using QSAR/QSPR approaches using existing experimental data [18, 36, 37, 40–43]. The QSAR concept is based on the congenericity principle, which hypothesizes that similar structures have similar properties and exhibit similar biological activities [44–47]. However, not all QSAR models are suitable for regulatory purposes because most use proprietary data and algorithms or lack documentation and transparency.

Several modeling guidance documents have been published [29, 48–52] to support the use of QSAR models in regulatory decision. In this study, OECD principles for building robust QSAR models were followed, if possible. The five OECD principles were: a defined endpoint; an unambiguous algorithm; a defined applicability domain (AD); appropriate measures for goodness-of-fit, robustness, and predictivity; and a mechanistic interpretation, if possible. This study, focused on development of QSAR/QSPR models for physicochemical properties, primarily using data from the publicly available PHYSPROP database [53] consisting of a set of 13 common physicochemical properties and environmental fate endpoints (Table 1).

**Table 1:** Endpoint datasets in the PHYSPROP database

Property abbreviation	Property	Source SD file
AOH	Atmospheric hydroxylation rate	EPI_AOP_Data_SDF.sdf
BCF	Bioconcentration factor	EPI_BCF_Data_SDF.sdf
BioHL	Biodegradability half-life	EPI_BioHC_Data_SDF.sdf
BP	Boiling point	EPI_Boil_Pt_Data_SDF.sdf
HL	Henry’s Law constant	EPI_Henry_Data_SDF.sdf
KM	Fish biotransformation half-life	EPI_KM_Data_SDF.sdf
KOA	Octanol–air partition coefficient	EPI_KOA_Data_SDF.sdf
KOC	Soil adsorption coefficient	EPI_PCKOC_Data_SDF.sdf
logP	Octanol–water partition coefficient	EPI_Kowwin_Data_SDF.sdf
MP	Melting point	EPI_Melt_Pt_Data_SDF.sdf
RB	Readily biodegradable	EPI_Biowin_Data_SDF.sdf
VP	Vapor pressure	EPI_VP_Data_SDF.sdf
WS	Water solubility	EPI_Wskowwin_Data_SDF.sdf

In this study, every endpoint was well defined, with documented sources and data curated from the publicly available PHYSPROP database [53–55]. In addition, genetic algorithms (GA) were employed during the variable selection step to identify a minimum number of the most suitable descriptors for each endpoint [56–58]. A weighted k-nearest neighbor (kNN) approach was used for model fitting to make the models as simple as possible [59, 60]. Goodness-of-fit, robustness, and predictivity were evaluated using internal fivefold cross-validation (CV) and external test set techniques [51, 61, 62]. The AD of the developed models were defined using local five-nearest neighbor and global leverage approaches [63–65]. The mechanistic associations between the descriptors and the endpoint being predicted were investigated and provided in QSAR model reporting format reports (QMRF) and registered in the European Commission’s Joint Research Center (JRC) QMRF Inventory [66, 67].

All models are freely available as an open-source, command-line application called OPERA (OPEn structure–activity/property Relationship App) [68, 69]. For transparency, all curated data used for training and testing as well as the QMRF documentation for each model are available in the Additional file 1: S1, a GitHub repository, ResearchGate, and the JRC’s QMRF Inventory [67, 69–82]. The OPERA models were used to predict properties for about 750,000 organic chemicals from the Distributed Structure-Searchable Toxicity (DSSTox) database and made publicly available, along with the experimental data, detailed prediction reports, and JRC validated QMRFs, through the EPA’s CompTox Chemistry Dashboard at https://comptox.epa.gov/dashboard/ [83, 84].

## Methods

### Datasets

Although there has been a dramatic increase in the number of data collections available for QSAR modeling over the last decades, the quality of the chemical structure information and associated experimental data remains of concern [85–88]. For the purpose of this modeling study, extensive curation work was conducted on 13 publicly available PHYSPROP physicochemical property and environmental fate datasets as previously reported [53, 54]. Data quality is important for QSAR/QSPR models and their predictive ability, as been demonstrated in previous work using the logP dataset which showed improved performance after curation [54]. The curation and correction of errors in the structure and identity of chemicals was performed using an automated workflow developed using the Konstanz Information Miner (KNIME), a free open-source data analytics, reporting, and integration platform [89].

As a first step in data curation, the workflow identified and corrected (when possible) errors and mismatches in chemical structure formats and identifiers (chemical names, Chemical Abstracts Service Registry Numbers [CASRN], Simplified Molecular Input Line Entry Specification [SMILES], and MOL), and various structure validation issues, including hypervalency and stereochemistry descriptions [90–93]. Data quality then was rated on a scale of 1–4, and only the top 2 classes (annotated as 3- and 4-star classes) were used as the model training data as explained in Mansouri et al. [54].

During the second step, QSAR-ready structures were generated from the high-quality chemical structure and property data using a KNIME standardization workflow developed previously [6, 94, 95]. The QSAR-ready workflow decreases the number of structures through the removal of duplicates generated by the standardization procedure. The standardization procedure includes removal of salt counterions (while retaining salt information in a separate field for potential later use), removal of stereochemistry, standardization of tautomers and nitro groups, correction of valences, neutralization of structures when possible, and removal of duplicates, among other steps, based on the International Chemical Identifier (InChI) code of the QSAR-ready structure. Due to its importance for melting point and boiling point endpoints, information regarding salts was considered, together with the QSAR-ready InChI code, during the duplicates removal step of these two specific datasets (see “Discussion”).

During the third step, modeling, the average experimental value was used if the difference between the duplicates was not significantly high (based on the standard deviation of the whole dataset). Otherwise, both duplicates were considered outliers and removed. Table 2 summarizes the evolution of the number of chemicals for the 13 datasets over the three steps.

**Table 2:** Numbers of chemicals associated with PHYSPROP datasets before and after curation and QSAR-ready standardization workflows

Property	No. of chemicals in dataset	No. of top-quality chemicals^a^	No. of QSAR-ready chemicals^a^
AOH	818	818 (100%)	745 (91.1%)
BCF	685	618 (90.2%)	608 (88.7%)
BioHL	175	151 (86.3%)	150 (85.7%)
BP	5890	5591 (94.9%)	5436 (92.3%)
HL	1829	1758 (96.1%)	1711 (93.5%)
KM	631	548 (86.8%)	541 (85.7%)
KOA	308	277 (90%)	270 (87.7%)
KOC	788	750 (95.2%)	735 (93.3%)
LogP	15,806	14,544 (92%)	14,041 (88.8%)
MP	10,051	9120 (90.7%)	8656 (86.1%)
RB	1265	1196 (94.5%)	1171 (92.5%)
VP	3037	2840 (93.5%)	2716 (89.4%)
WS	5764	4372 (75.8%)	4224 (73.3%)

^a^Percentages relative to the original dataset shown in parentheses; 2D descriptors only used

### Descriptor calculation

The curated chemical structures were used to calculate molecular descriptors using the free and open-source software PaDEL [96]. PaDel was used to calculate only 1D and 2D descriptors; 3D descriptors were avoided even though they could potentially add useful chemical information about the molecules [27, 97]. We decided to use only 2D descriptors to keep the models as simple as possible, to speed up predictions, and to avoid repeatability problems associated with 3D descriptor values. These can arise due to differences between conformers, especially with very flexible molecules requiring geometry optimization. These differences can affect the predictability of the resulting chemical properties [98, 99]. To avoid inconsistencies due to explicit hydrogen atoms and interpretation of aromatic rings by the software during descriptor calculations, the aromaticity option was set to auto-detection as suggested by the PaDEL developers to fix known issues [100]. The need for the auto-detection setting was verified by performing tests that confirmed that PaDEL can interpret aromaticity in different ways for the same chemical, depending on whether it is provided in MOL, SMILES, or SDF format, and can provide different values for certain descriptors, such as number of aromatic rings.

A total of 1444 molecular descriptors were calculated, including constitutional, topological, functional group counts; fragmental, atom-type E-state indices; and other physicochemical descriptors. To reduce collinearity among descriptors, a correlation filter with a threshold of 0.96 was applied. For each pair of descriptors with a correlation coefficient higher than the threshold, the one showing the largest pair correlation with all the other descriptors was excluded. Then, descriptors with constant, near-constant (using a standard deviation of 0.25 as a threshold), or at least one missing value were removed. The remaining reduced sets ranging from 800 to 1000 descriptors were used for subsequent modeling analysis.

### Fitting algorithm

Several model-fitting techniques have been used in the literature to model physicochemical properties and biological activity endpoints [101–106]. The application of these methods, based on different mathematical strategies with varying degrees of complexity, aims to explore chemical space and balance potential biases inherent in each single modeling algorithm. However, the increase in model complexity is not always justified with statistically significant increases in predictive accuracy [107, 108]. Because the goal of this study is to facilitate the interpretability of the models (a requirement of regulators according to OECD guidelines), one of the simplest yet highly reliable methods, kNN, was selected [59, 60, 103, 109]. This method can be applied to both quantitative and qualitative data and is very similar to read-across, a widely used method in the regulatory field [110, 111].

The kNN method was applied to estimate the best relationship between chemical information, encoded in molecular descriptors, and the modeled activity of chemicals based on the closest chemicals to the query chemical. Its classification rule is conceptually quite simple: each predicted chemical is classified according to the majority of its k nearest neighbors in the selected descriptor space of the training set. In this study, the classical kNN classification algorithm has been refined so that the contribution of each of the k neighbors is weighted according to distance to the query point, giving greater weight to closer neighbors [18, 112]. The weighted kNN algorithm uses the Euclidean metric to measure distances between molecules. The Euclidean distance was calculated using the auto-scaled descriptor values [113, 114]. For each dataset, first the training set was scaled, and its parameters saved. Then, the test set was scaled using the same parameters. Even with this refinement, the weighted kNN is an unambiguous algorithm that fulfills the transparency requirements of OECD principle 2, with an optimal compromise between model complexity and performance.

### Variable selection

Variable selection techniques are usually applied to find the optimal subset with a minimum number of molecular descriptors [57, 115]. This step consisted of coupling GA with the weighted kNN algorithm, and was applied in fivefold CV on the auto-scaled training set (75% of each dataset). GA starts from an initial random population of chromosomes, which are binary vectors representing the presence or absence of the molecular descriptors [56–58]. An evolutionary process is simulated to optimize a defined fitness function, and new chromosomes are obtained by coupling the chromosomes of the initial population with genetic operations (crossover and mutation). This evolution process was repeated 100 times for each of the 100 consecutive independent runs, with a 0.01 probability of mutation and a 0.5 probability of crossover on 30 chromosomes. The generations of populations are evaluated and ranked during the evolution process based on goodness-of-fit functions used to optimize the models and calculated in CV, maximizing the accuracy and minimizing the number of descriptors. The number of neighbors (k) was optimized within the range of 3–7. The k value giving the lowest classification error in CV was selected as the optimal value. The descriptors were then ranked based on frequency of selection during the GA runs, and the final set of descriptors encoding the most relevant structural information to the modelled endpoint was picked in a forward-selection manner. If the algorithm did not converge during the first 100 runs, another round of 100 runs was performed on the top performing half of the initial set of descriptors, ranked by frequency of selection during the first 100 runs.

### Validation methods

**Fig. 1:**
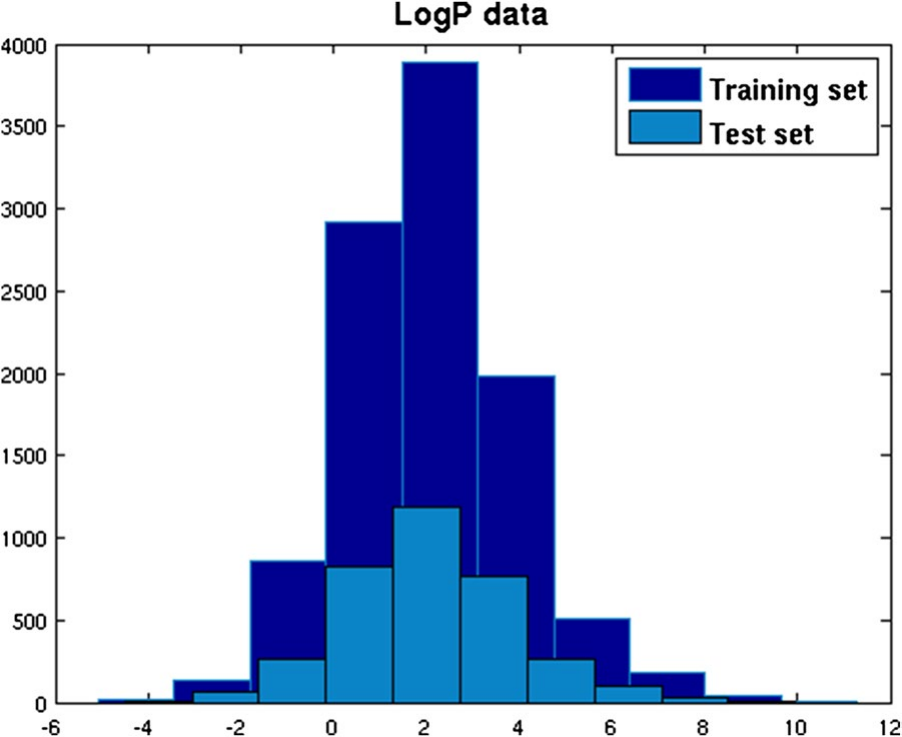
Distribution of experimental logP values between training and test sets

Each of the 13 datasets was randomly divided into training and test sets containing 75 and 25% of the total number of considered molecules, respectively. Selection was performed maintaining a distribution of the quantitative data values and class proportions for the qualitative data. The outcome was that the number of test molecules for each range/class was proportional to the number of training molecules of that range/class. Figure 1 shows the distribution of logP values across the training and test sets. Figures similar to Fig. 1 were generated for the full set of models and are provided in the Additional file 1: S1. These figures can be viewed in the calculation reports on the CompTox Chemistry Dashboard [84] (https://comptox.epa.gov/dashboard).

The training set was used to select molecular descriptors and to build the models. Molecules within the test set were used to evaluate the predictive ability of the built models. Fivefold CV was used during model optimization and descriptor selection. This procedure is similar to constantly dividing the initial set into training and test sets, containing 80 and 20% of the total number of chemicals, respectively.

### Model performance

This study used two types of models, a classification model for the RB dataset and continuous models for the other 12 datasets. The performance of each type of model was evaluated as summarized below.

#### Classification model

The performance of the classification model was evaluated using sensitivity (Sn), the true positive rate, and specificity (Sp), the true negative rate. These statistical indices represent the ability of the model to correctly predict two classes, such as active and inactive molecules (readily biodegradable and non-readily biodegradable) [59, 116]. These indices are calculated from the confusion matrix, which collects the number of samples of the observed and predicted classes in the rows and columns, respectively [117]. The classification parameters are defined using the number of true positives (TP), true negatives (TN), false positives (FP) and false negatives (FN).

The most important parameter considered during the evaluation step was the balanced accuracy (BA), usually expressed as a fraction calculated as follows:

1 $$BA = \frac{{\left({Sn + Sp} \right)}}{2}$$

where the Sn is calculated as follows:

2 $$Sn = \frac{TP}{TP + FN}$$

and the Sp is calculated as follows:

3 $$Sp = \frac{TN}{TN + FP}$$

In the case of two-class models, the Sn of one class corresponds to the Sp of the other class. These indices were used to better estimate performance of the classification model in the presence of a dataset with an unequal number of molecules in each class. In this study, BA, Sn, and Sp are expressed as ratios and not as percentages.

#### Continuous models

The quality of continuous models was evaluated using two groups of statistical indices, goodness-of-fit parameters and goodness-of-prediction parameters. Goodness-of-fit parameters measure the fitting ability and are used to measure the degree to which the model is able to explain the variance contained in the training set [118]. Traditionally, regression model quality is evaluated using the root mean square error (RMSE) calculated as the root of the average of the residual sum of squares:

4 $$RMSE = \sqrt {\frac{{\mathop \sum \nolimits_{i = 1}^{n} \left( {y_{i} - \hat{y}_{i} } \right)^{2} }}{n}}$$

where n is the number of training compounds, and $\hat{y}_{i}$ and $y_{i}$ are the estimated and observed responses, respectively.

The coefficient of determination *R*^*2*^ is a useful parameter because it is independent from the response scale, contrary to RMSE. (RMSE is in turn useful because it provides an estimate of the expected error magnitude on the scale of the property being calculated.) It is the square multiple correlation coefficient calculated as follows:

5 $$R^{2} = \frac{{\mathop \sum \nolimits_{i = 1}^{n} (\hat{y}_{i} - y_{i} )^{2} }}{{\mathop \sum \nolimits_{i = 1}^{n} \left( {y_{i} - \bar{y}} \right)^{2} }}$$

where $\hat{y}_{i}$ and $y_{i}$ are the estimated and observed responses, respectively, and $\bar{y}$ is the average observed response over the n training compounds.

Goodness-of-prediction parameters measure the true predictive ability of a model and are related to the reliability of prediction. These parameters are used in the validation step. The most important parameters are the root mean square error in prediction (RMSEP) and the predictive squared correlation coefficient Q^2^. RMSEP is calculated as follows:

6 $$RMSEP = \sqrt {\frac{{\sum_{i = 1}^{{n_{EXT} }} \left( {y_{i} - \hat{y}_{i} } \right)^{2} }}{{n_{EXT} }}}$$

where $n_{EXT}$ is number of test compounds, and ${\hat{y}}_{i}$ and ${y}_{i}$ are the estimated and observed responses respectively.

Different ways of calculating Q^2^ are available in the literature [50, 61, 62]. However, because RMSEP (and RMSE) depends on the scale reference, Q^2^ must fulfill the ability of R^2^ to be independent of the response scale [51]. Also, to be a subjective representative of the true predictivity of a model, Q^2^ must be invariant for a fixed RMSEP value, be invariant to the splitting of the external data into subsets (ergodic principle), and correlate perfectly with RMSEP. This study used the Q^2^ formula below demonstrated by Todeschini et al. [51] because it is the only formula that fulfils all these requirements.

7 $$Q^{2} = 1 - \frac{{\sum\nolimits_{i = 1}^{{n_{EXT} }} {{{\left( {y_{i} - \hat{y}_{i} } \right)^{2} } \mathord{\left/ {\vphantom {{\left( {y_{i} - \hat{y}_{i} } \right)^{2} } {n_{EXT} }}} \right. \kern-0pt} {n_{EXT} }}} }}{{\sum\nolimits_{i = 1}^{{n_{TR} }} {{{\left( {y_{i} - \bar{y}} \right)^{2} } \mathord{\left/ {\vphantom {{\left( {y_{i} - \bar{y}} \right)^{2} } {n_{TR} }}} \right. \kern-0pt} {n_{TR} }}}}}$$

where $n_{EXT}$ and $n_{TR}$ are the numbers of test and training compounds, respectively, and $\hat{y}_{i}$ and $y_{i}$ are the estimated and observed responses, respectively.

### Applicability domain and reliability assessment

The modeling approach used in this study is applicable to heterogeneous collections of organic chemicals. As a result of the implementation of the models, several pieces of information are provided to help the user evaluate the reliability of a prediction. The chemical structure is first assessed to see if it falls within the AD of the training set chemical space. Then, the accuracy of the predicted value is reported based on the accuracy of prediction of the neighboring chemicals in the training set using a leave-one-out procedure, weighted by similarity to the query chemical. This approach fulfills the requirements of the third OECD principle by defining the limitations in terms of the types of chemical structures, physicochemical properties, and mechanisms of action for which the model can generate reliable predictions.

The AD of the model is assessed at two independent levels using two different distance-based methods. First, a global AD is determined using a leverage approach that checks whether the query structure falls within the multidimensional chemical space of the whole training set [63]. The leverage of a query chemical is proportional to its Mahalanobis distance measured from the centroid of the training set [119, 120]. The leverages of a given n-chemical by p-descriptor matrix, X, are obtained from the diagonal values of the hat matrix, H, calculated as follows:

8 $${\text{H}} = {\text{X}}({\text{X}}^{\text{T}} {\text{X}})^{ - 1} {\text{X}}^{\text{T}}$$

This approach is associated with a threshold leverage that corresponds to 3 * p/n, where p is the number of model variables (descriptors) and n is the number of training compounds. A query chemical with leverage higher than the threshold is considered outside the AD and can be associated with unreliable prediction.

The leverage approach has specific limitations, in particular with respect to gaps within the descriptor space of the model or at the boundaries of the training set. To obviate such limitations, a second tier of AD assessment was added. This is a local approach, which only investigates the vicinity of the query chemical. This local approach provides a continuous index ranging from 0 to 1, which differs from the first approach that provides only Boolean answers (yes or no). This local AD index is relative to the similarity of the query chemical to its five nearest neighbors in the p-dimensional space of the model using a weighted Euclidean distance. The higher this index, the more the prediction is expected to be reliable.

These two AD methods are complementary and can be interpreted as summarized below.

- If a chemical is considered outside the global AD and has a low local AD index (< 0.4), the prediction can be unreliable.
- If a chemical is considered outside the global AD but the local AD index is average (0.4–0.6), the query chemical is on the boundary of the training set but has quite similar neighbors (average reliability). If the local AD index is high (> 0.6), the prediction can be trusted.
- If a chemical is considered inside the global AD but the local AD index is average (0.4–0.6), the query chemical falls in a “gap” of the chemical space of the model but still falls within the boundaries of the training set and is surrounded with training chemicals. The prediction therefore should be considered with caution.
- If a chemical is considered inside the global AD and has a high local AD index (> 0.6), the prediction can be considered reliable.

A confidence level index also was calculated based on the accuracy of the predictions of the five nearest neighbors weighted by their distance to the query chemical. This index gives the user an estimate regarding the reliability of the prediction when the query chemical is inside the AD. Further details about the implementation of AD approaches can be found in Sahigara et al. [63].

### Software and calculations

Data-mining steps, including structures and experimental data pre-treatment, QSAR-ready data preparation, and training/test set splitting were performed using KNIME (version 3) [89]. Molecular descriptors were calculated using PaDEL software (version 2.21) from QSAR-ready structures in SDF files [96]. All modeling steps and calculations, including GA variable selection, model fitting, and validation as well as AD and accuracy assessment were performed using MATLAB (version 8.2, glnxa64) [121].

## Results

### Descriptor selection and model fitting

The curation step performed during previous work [54] helped in the selection of the highest quality data from the publicly available PHYSPROP [53] database for the 13 available physicochemical property and environmental fate datasets (Table 1). The resulting validated chemical structures were used to calculate PaDEL 1D and 2D descriptors (a total set of 1444). Although certain filters were applied (collinearity, missing values, and constant and near-constant), large numbers of descriptors (800–1000 across all datasets) remained available for modeling. To include only the most pertinent descriptors in the QSAR models, the variable selection procedure was performed on training chemicals (75% of the data) in two subsequent steps. The initial 100 independent GA runs were conducted on the full list of the descriptors associated with each dataset, then a second set of 100 independent GA runs were conducted on the 50% of descriptors that showed the highest frequency of selection during the first round. This two-step approach was adopted in order to ensure the convergence of the selection towards the same final subset with the highest frequency of selection. The subsets of molecular descriptors yielding the highest model performance were selected at the end of the second round of GA (forward step selection based on decreased frequency of selection), and were used to fit and calibrate the final models. The final models were selected by considering a minimum number of descriptors and keeping a balance between statistics in fitting and in fivefold CV. This procedure has been shown to minimize the risk of overfitting [58, 61, 112].

The QSAR models were validated using the test set molecules, which did not participate in the descriptor selection and model fitting and calibration steps. Training and test sets for all OPERA models are provided in the Additional file 1: S1.

### Models and performance

Table 3 summarizes the performance of the selected models.

**Table 3:** Performance of the selected models in fitting, CV, and on the test sets

Property	No. of descriptors	Fivefold CV (75%)		Training (75%)			Test (25%)		
		Q^2^	RMSE	Dataset	R^2^	RMSE	Dataset	R^2^	RMSEP
AOH	13	0.85	1.14	516	0.85	1.12	176	0.83	1.23
BCF	10	0.84	0.55	469	0.85	0.53	157	0.83	0.64
BioHL	6	0.89	0.25	112	0.88	0.26	38	0.75	0.38
BP	13	0.93	22.46	4077	0.93	22.06	1358	0.93	22.08
HL	9	0.84	1.96	441	0.84	1.91	150	0.85	1.82
KM	12	0.83	0.49	405	0.82	0.5	136	0.73	0.62
KOA	2	0.95	0.69	202	0.95	0.65	68	0.96	0.68
KOC	12	0.81	0.55	545	0.81	0.54	184	0.71	0.61
LogP	9	0.86	0.69	10,537	0.86	0.67	3513	0.86	0.78
MP	16	0.74	50.20	6486	0.75	49.12	2167	0.74	52.27
VP	12	0.91	1.08	2034	0.91	1.08	679	0.92	1
WS	11	0.87	0.81	3158	0.87	0.82	1066	0.86	0.86

Property	Descriptor	BA	Sn–Sp	Dataset	BA	Sn–Sp	Dataset	BA	Sn–Sp
RB	10	0.8	0.82–0.78	1197	0.8	0.82–0.79	411	0.79	0.81–0.77

The continuous models yielded Test R^2^ in the range of 0.71–0.96. For most of the models the external R^2^ and the internal Q^2^ are close in value, which indicates that overfitting has not occurred. The exceptions are BioHL (0.89–0.75), KM (0.83–0.73) and KOC (0.81–0.71). The drop in performance for these properties could be due to the biological complexity of these endpoints compared to the physicochemical properties. The final models use small numbers of descriptors which helps with model transparency and facilitates mechanistic interpretation, as required by OECD principles 2 and 5. Indeed, the number of descriptors ranged from only 2 descriptors for KOA to 16 descriptors for MP, with an average of about 10 descriptors. The RB model, a classification model, also shows the same robustness as the continuous models, with an additional characteristic that is the balance between the Sn and Sp parameters, indicating that the model is as good at predicting readily biodegradable molecules versus non-readily biodegradable molecules.

### Implementation of the models in OPERA

All 13 models were implemented in MATLAB and compiled into OPERA, a standalone command-line application for Microsoft Windows and Linux [68, 69]. This application uses an input file containing one or multiple QSAR-ready structures in SMILES strings or MOL or SDF format. (A QSAR-ready workflow will be implemented in a future version of OPERA.) After parsing and checking the structures, OPERA calculates the necessary descriptors for the requested models using the embedded PaDEL software (version 2.21) with its developer’s recommended options for consistency [96, 100]. It then writes the requested results to a tab-delimited txt file or a comma-delimited csv file. The output file contains the OPERA predictions, AD and accuracy assessment, and up to five nearest neighbors from the training set. The neighbors are identified by their CASRNs, QSAR-ready InChI keys, and a unique DSSTox database substance identifier (DTXSID) that links them to the CompTox Chemistry Dashboard [84]. All these details are provided in the output of single chemical and batch mode calculation. However, the CompTox Dashboard provides AD and accuracy assessment for one chemical per page but in batch mode downloading provides predictions only [84, 122]. Pre-calculated PaDEL descriptors can also be used as inputs to avoid re-calculating them, which can be time-consuming for large files (such as the DSSTox database of over 700K structures). The users are given different options for both input and output to allow for additional flexibility. The available input/output options and usage arguments are described in a help file provided as Additional file 2: S2.

OPERA executables (current version 1.5), C/C++ libraries, and the associated MATLAB source code are available for free on Github under the Massachusetts Institute of Technology (MIT) license (https://github.com/kmansouri/OPERA.git) together with the data and QMRFs (Table 4) that are also available in the Additional file 1: S1 and on the JRC repository [67].

**Table 4:** The QMRF reports published online

Property	JRC report ID	DOI
AOH	Q17-22b-0024	10.13140/RG.2.2.24685.59368/2
BCF	Q17-24a-0023	10.13140/RG.2.2.17974.70722/1
BioHL	Q17-23b-0022	10.13140/RG.2.2.34751.92320/1
BP	Q17-12-0021	10.13140/rg.2.2.33074.20160/1
HL	Q17-19-0020	10.13140/rg.2.2.17764.99201/1
KM	Q17-66-0019	10.13140/rg.2.2.31186.76482/1
KOA	Q17-18-0018	10.13140/rg.2.2.14409.54883/1
KOC	Q17-26-0017	10.13140/rg.2.2.27831.32163/1
LogP	Q17-16-0016	10.13140/rg.2.2.12731.82723/1
MP	Q17-11-0015	10.13140/rg.2.2.26153.60003/1
RB	Q17-23a-0014	10.13140/rg.2.2.19442.71369/1
VP	Q17-14-0013	10.13140/rg.2.2.32864.48641/1
WS	Q17-13-0012	10.13140/rg.2.2.16087.27041/1

### OPERA applied to the CompTox Chemistry Dashboard

The curation of PHYSPROP datasets and the development of the OPERA models were part of the CompTox Chemistry Dashboard project [84]. The CompTox Chemistry Dashboard is a web-based application and data hub developed by EPA’s NCCT [83]. Chemical substances surfaced via the Dashboard are hosted in the underlying DSSTox database with associated identifiers (such as CASRNs, systematic and common names, and other chemical structure identifiers, including InChIs and SMILES strings). The Dashboard is used to search the DSSTox database using a simple alphanumeric text entry box accessible on the home page [83]. A successful search result will result in a chemical page header that provides the following [123]:

- Chemical structure image (with the ability to download in MOL file format).
- Intrinsic properties (such as molecular formula and monoisotopic mass).
- Chemical identifiers (such as systematic name, SMILES string, InChI string, and InChIKey).
- Related compounds (based on molecular skeleton search, molecular similarity search, and presence of the chemical in various mixtures and salt forms).
- List of databases in which the chemical is present (such as ToxCast and Tox21).
- Record citation, including the unique DTXSID.

**Fig. 2:**
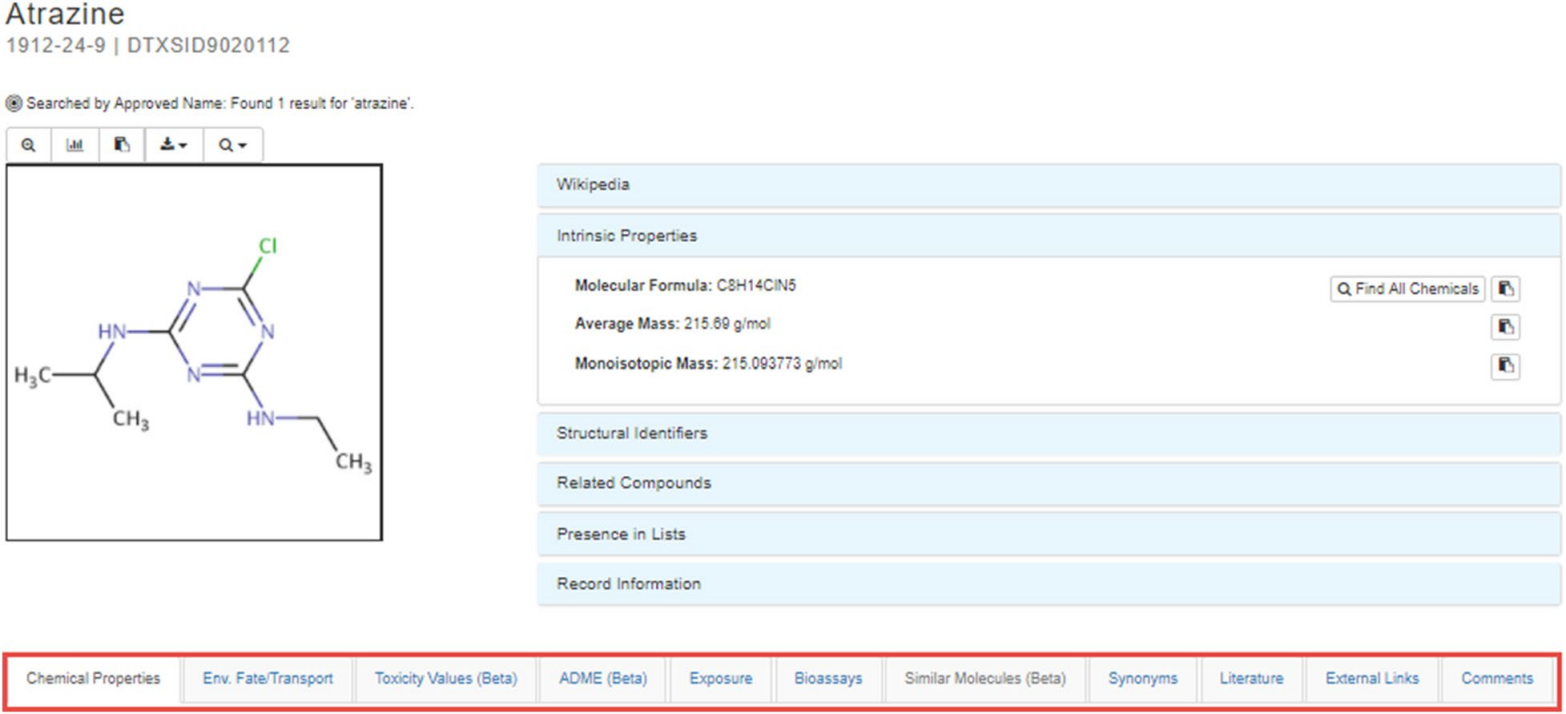
Results search header for atrazine on the CompTox Chemistry Dashboard

Figure 2 shows a search chemical page header for atrazine.

**Fig. 3:**
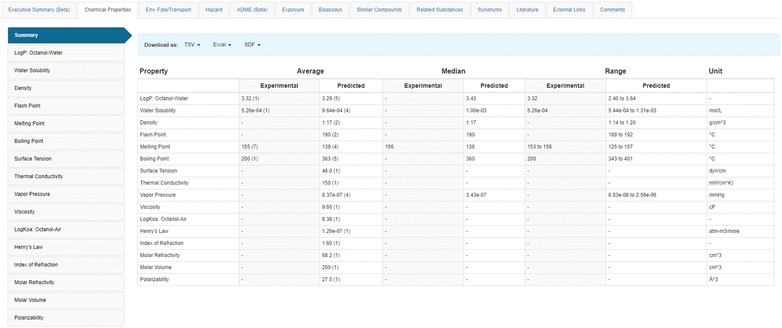
Summary view of experimental and predicted physicochemical properties

Below the header are a series of data tabs (shown in the red box in Fig. 2). The “Chemical Properties” tab (expanded in Fig. 3) and Environmental Fate and Transport tabs contain experimental properties assembled from various sources and properties predicted by a series of algorithms, including (1) ACD/Labs predicted data sourced from Open PHACTS [124]; (2) EPI Suite data sourced from [53]; (3) NICEATM predictions for a small number of properties [37]; and (4) OPERA predicted data, discussed in more detail below.

**Fig. 4:**
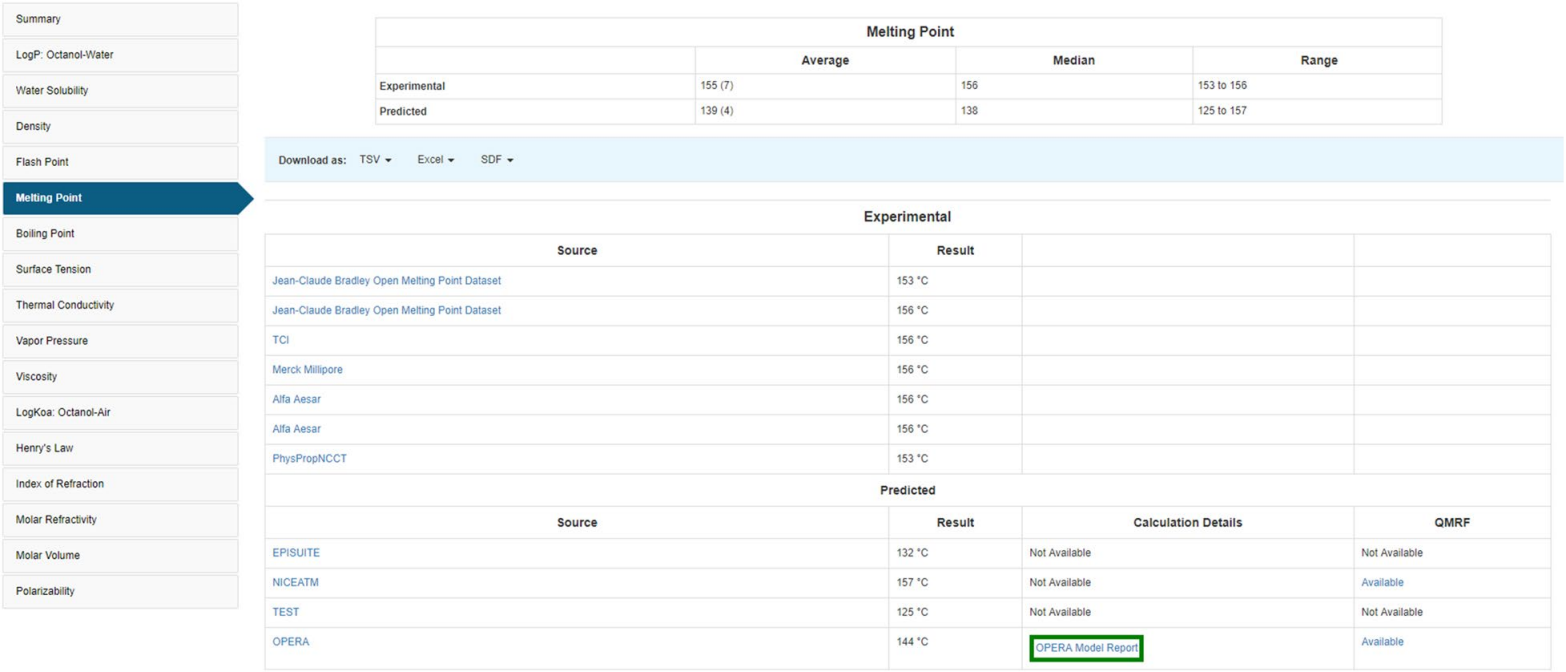
Melting Point (MP) experimental and predicted values from different sources

The experimental and predicted chemical properties data tables show the average, median, and range of properties associated with a particular chemical (Fig. 4).

**Fig. 5:**
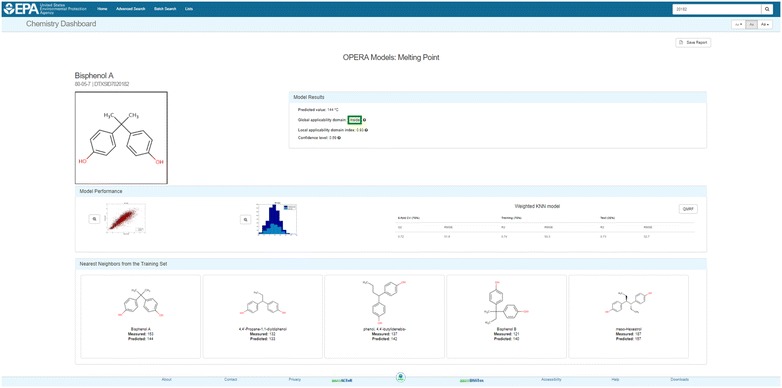
OPERA prediction calculation report for the melting point of bisphenol A

Both National Toxicology Program Interagency Center for the Evaluation of Alternative Toxicological Methods (NICEATM) and OPERA QMRF reports are available as PDF files via a hyperlink. In addition to the QMRFs [70–82], additional information about OPERA predictions is provided in a detailed calculation report (Fig. 5), which adds another level of transparency by showing the global performance of the models, the AD, and the reliability assessment. It also provides up to five nearest neighbors from the training set (where available), with their experimental and predicted values as an additional reliability assessment for the user.

A batch search allows users to input search lists, including chemical names, CASRNs, and InChI Keys, and to retrieve formulae, masses, DTXSIDs, and other data related to chemical bioactivity and exposure, including the download of data associated with OPERA predictions as either tab-separated values or Excel or SDF files. An example downloaded Excel table with predicted OPERA values is provided as Additional file 3: S3.

A detailed help file regarding how to use the Dashboard is available online (https://comptox.epa.gov/dashboard/help). Various subsets of data associated with the Dashboard are available as open data and can be obtained from the downloads page (https://comptox.epa.gov/dashboard/downloads). The download page also provides access to a zip file containing training and test data sets associated with the OPERA models and the KNIME workflows used for the original curation of the PHYSPROP data.

## Discussion

### OPERA logP modeling

The QSAR community has extensively modeled multiple physicochemical properties, such as logP, using different approaches [38, 41, 125–128]. Most of these published models are reported with R^2^ for fitting and R^2^/Q^2^ validation within a range of 0.8–0.9. However, the possibility of objective comparisons is undermined by the absence of standardized metrics and evaluation equations as well as the lack of transparency in training and test sets of chemicals and data, AD, descriptors, and code or executables. This study attempts to deliver transparency in terms of access to data and model performance statistics. The classical approach of comparing models by global R^2^/Q^2^ fitting performance may or may not reflect higher predictive ability, especially when dealing with different sizes of datasets, for example. Therefore, comparisons of model fit should be local and specific, not based on overall statistics. Also, every model, even though it may be built correctly and validated, has its own strengths and limitations [129]. A model should include tools that can help assess the reliability of its predictions. A model can be evaluated locally only within its AD, which is the interpolation space of the training set [63, 64]. Any extrapolation outside of that specific area of structure space is most likely unreliable.

**Fig. 6:**
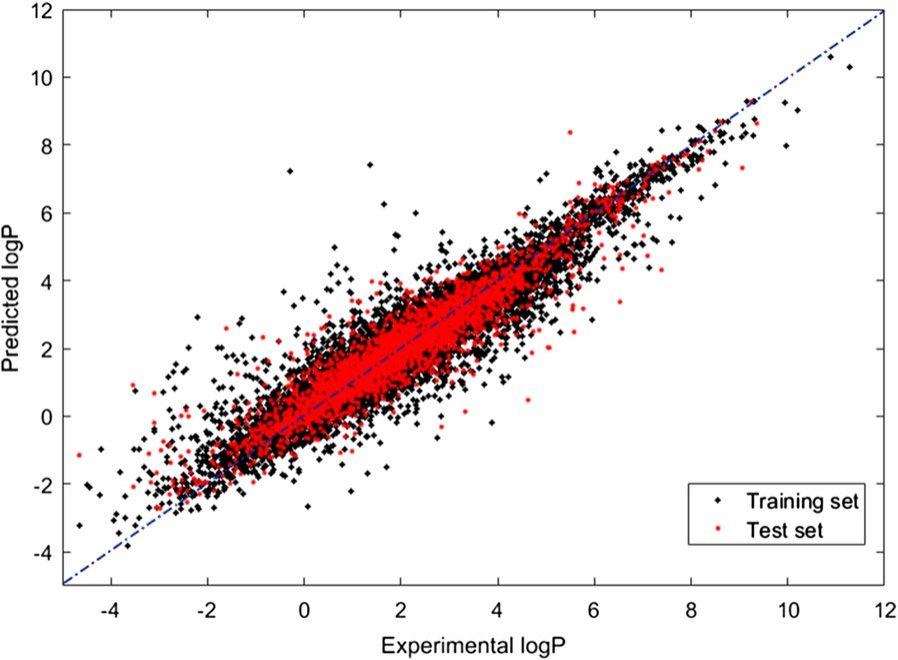
Experimental and predicted values for training and test set of OPERA logP model

The logP model presented in this study showed good overall performance and, more importantly, also demonstrated stable statistics across the different steps of modeling and validation (Table 3). This indicates that the model is both robust and reliable. Figure 6 presents the experimental and predicted values for the training and test sets for logP.

In addition, the OPERA logP model is developed to compromise between model complexity and predictive ability, which are OECD recommendations for QSAR models developed for regulatory applications. This model is built using an unambiguous weighted kNN algorithm and uses only nine descriptors (variables). Figures similar to Figs. 1 and 6 were generated for the full set of models and are provided in the Additional file 1: S1 as well as the calculation reports on the CompTox Chemistry Dashboard [83, 84].

The OPERA logP model performance was evaluated in relation to a reference model, EPI Suite’s KOWWIN logP model. This model was chosen because the OPERA training set uses curated data derived from the PHYSPROP database, which in its original form was used to develop the KOWWIN logP model. The exact training subset used to develop the EPI Suite KOWWIN model and the AD for the KOWWIN model are not fully known. Thus, the comparison was not based on overall training, CV and test set but was performed locally for a specific subset of the data.

**Fig. 7:**
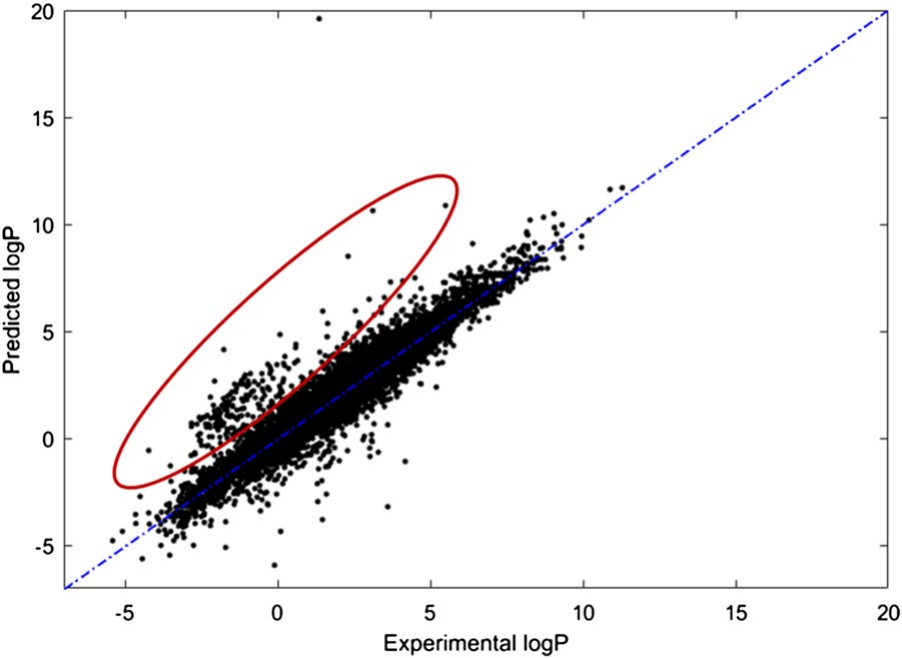
LogP predictions for KOWWIN model. The overestimated cluster selected for comparison is highlighted in a red ellipse

To show localized improvement of the OPERA logP model compared to the KOWWIN logP model, a small subset of data (280 chemicals) was selected for which the KOWWIN logP model overestimates the values (represented by the red stars in Fig. 7).

**Fig. 8:**
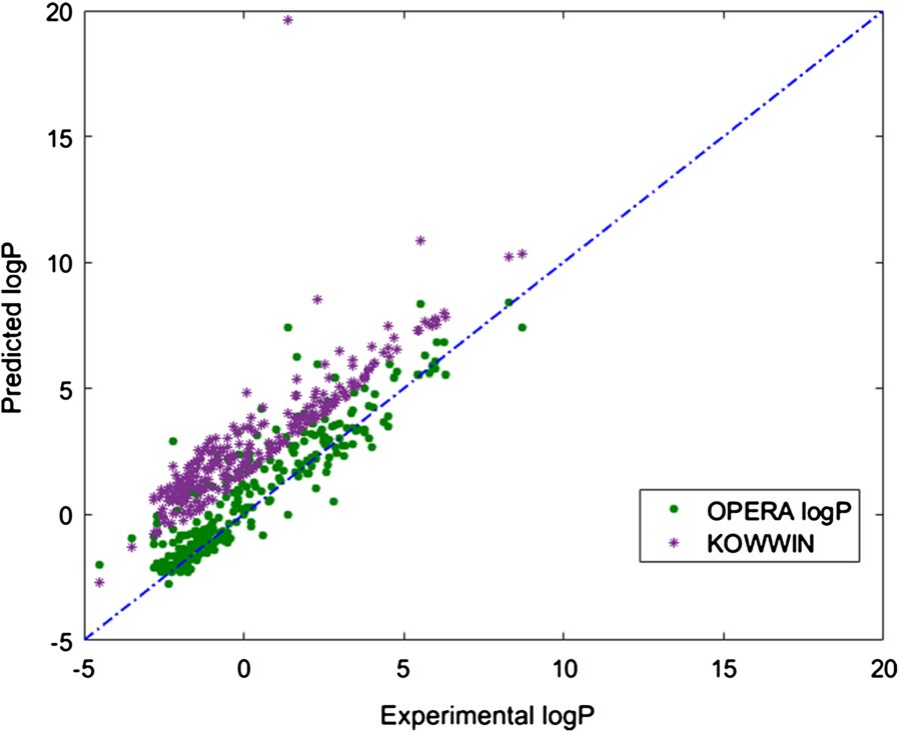
LogP predictions for KOWWIN model in purple stars compared to OPERA model in green circles

Figure 8 shows that the OPERA model provides estimations of logP closer to observed values than the EPI Suite KOWWIN model.

An investigation of the chemical space represented by the cluster in red was a specific family of chemicals. However, the 280 chemicals tested turned out to be heterogeneous, with no obvious common structural features. It is possible that these chemicals are outside of the AD of KOWWIN’s training set but inside the AD of the OPERA logP model, which is built on a newer version of the PHYSPROP database and possibly associated with a larger AD. The comparison shows that different models can show similar global statistics but provide very different predictions for certain local regions of chemical space and local ADs. Here, for this specific subset, R^2^ and RMSE for KOWWIN were − 0.35 and 2.79, respectively compared to an R^2^ equal to 0.75 and an RMSE of 1.19 for OPERA logP (Table 5). Such a difference in prediction performance, even though it is significant for this specific group of chemicals, does not make a difference in the global statistics of a large dataset (280 vs ~ 10,000 chemicals).

**Table 5:** Local comparison of OPERA logP and KOWWIN

Model	R^2^	RMSE
OPERA logP	0.75	1.19
KOWWIN	− 0.35	2.79

Another example of improved OPERA model predictions for a local area of the chemical space is the logP data for nine polybrominated diphenyl ethers (PBDE) [130]. PBDEs (209 congeners) were commonly used as flame retardants but now are recognized for their toxicity, persistence, and potential for bioaccumulation and long-range atmospheric transport [131]. PBDEs are considered persistent organic pollutants and are prioritized in regulations [132–134]. As Table 6 shows, the predicted logP values for a number of PBDEs were underestimated in older versions of the OPERA model. After retraining of the models using experimental data, the new predictions are more accurate for these nine PBDEs and therefore are hypothesized to be more accurate for the remaining 200 congeners. Other congeners, such as BDE-104 (DTXSID60879916) are predicted within the global and local AD (0.64) with a high confidence level (0.78) [135]. Even congeners considered outside the global AD, such as BDE-150 (DTXSID80879953), are associated with an acceptable local AD index (0.62) and high confidence level (0.78) [136]. This last example shows the advantage of using two approaches for the AD (instead of a global one only) in addition to the confidence level in prediction that allows the user to make rational judgement about prediction reliability.

**Table 6:** Newly added data for PBDEs and resulting OPERA model predicted logP values

DTXSID	Name	CASRN	OPERA logP (old)	Newly added data	OPERA logP (new)
DTXSID40872703	BDE-17	147217-75-2	5.13	5.74 ± 0.22	5.80
DTXSID4052710	BDE-28	41318-75-6	4.17	5.94 ± 0.15	5.97
DTXSID3030056	BDE-47	5436-43-1	5.65	6.81 ± 0.08	6.56
DTXSID4052685	BDE-85	182346-21-0	6.00	7.37 ± 0.12	7.38
DTXSID9030048	BDE 99	60348-60-9	6.03	7.32 ± 0.14	7.38
DTXSID4052689	BDE-100	189084-64-8	6.04	7.24 ± 0.16	7.26
DTXSID4030047	BDE-153	68631-49-2	6.00	7.90 ± 0.14	7.72
DTXSID3052692	BDE-154	207122-15-4	5.94	7.82 ± 0.16	7.72
DTXSID8052693	BDE-183	207122-16-5	6.09	8.27 ± 0.26	8.19

Through the calculation reports associated with OPERA model predictions, the CompTox Chemistry Dashboard provides decision-makers specific quantitative and qualitative information on how much to trust a particular prediction [84]. The Dashboard enhances the transparency for the OPERA model predictions because it shows both the model strengths and limitations. Visual inspection of the data represented in the prediction reports reveals a number of compounds outside the AD (both global and local) and associated with a low confidence level, making the prediction for those compounds unreliable. One example compound is Irganox 1010 (DTXSID1027633), which in the OPERA logP model has a local AD index of only 0.11 and a confidence level of 0.2. This low confidence level indicates that the prediction should not be considered accurate [137]. The predicted logP value of 7.25 from the OPERA model seems to underestimate the value for this structure. Irganox 1010 had a measured logP value of 1.3 in the PHYSPROP logP training set but was considered an outlier and removed during the latest update of the Dashboard (released on August 11, 2017). Such chemicals with few to no neighbors in the calculation report (https://comptox.epa.gov/dashboard/dsstoxdb/calculation_details?model_id=22&search=27633) do not have enough representatives in the training sets of the models and indicate the limits of model reliability. This example also shows that the AD approaches and confidence levels are useful ways to expose the boundaries of the covered interpolation space of a model and therefore its reliability.

OPERA was recently compared with 7 other software applications in estimating logP, melting point, vapor pressure and water solubility for a dataset of polychlorinated biphenyls, polybrominated diphenyl ethers, polychlorinated dibenzodioxins, and polycyclic aromatic hydrocarbons and demonstrated the best performance for the prediction of logP and good performance across the other parameters [122].

### OPERA MP modeling with and without salts

Another benefit of the OPERA prediction reports on the Dashboard is consideration of the presence of salts in addition to the desalted QSAR-ready structures for MP estimation. The influence of salt counterions on melting points is an important consideration for this particular endpoint. (All of the other endpoints model the behavior of the salts in solution, where they are assumed to be largely ionized, so that the properties of the organic moiety will be independent of the counterion.) The OPERA model’s ability to consider the presence of salts shows that the selection of fit-for-purpose standardization workflows (such as the one used in this work [94, 95]) to generate QSAR-ready structures for specific endpoints is important. Adding information regarding the salt form increases the prediction accuracy of the models by considering the correct nearest neighbors. Two examples demonstrate the increased prediction accuracy, guanidine (DTXSID0023117) and guanidine monohydrochloride (DTXSID7058757). For guanidine, both the PHYSPROP database and another source (Jean-Claude Bradley dataset [138]) agree that the measured MP is 50 °C, while the MP of the salt form is 182 °C according to the PHYSPROP database [139, 140]. The OPERA model predicts the guanidine MP at 62.9 °C and displays unsalted neighbors on the prediction report [141]. However, for the salted form, guanidine monohydrochloride, the OPERA model predicts an MP of 182 °C, with only salted neighbors in the prediction report [142]. The NICEATM model [37] predicts both salted and unsalted forms to have a MP of 88.4 °C, which clearly significantly underestimates the MP of guanidine monohydrochloride.

The OPERA MP model can operate with and without salt information by considering the salt form as the 16th descriptor. To evaluate the impact of including and excluding this last descriptor on the statistics of the model, a comparison of the two modes was performed (Table 7).

**Table 7:** OPERA model prediction performance for MP with and without salt information

Mode	Variables	Fivefold CV (75%)		Training (75%)		Test (25%)	
		Q^2^	RMSE (°C)	R^2^	RMSE (°C)	R^2^	RMSEP (°C)
No salts	15	0.72	51.8	0.74	50.27	0.73	52.72
With salts	16	0.74	50.2	0.75	49.12	0.74	52.27

Table 7 shows a slight improvement of the statistics for the mode with salts information. But these *global statistics* are for the whole training and test sets and do not reflect the influence on the salted compounds, which represent less than 2% of the two datasets.

Table 8 shows the improvement of the MP statistics for salt-form chemicals only. This table compares the RMSE values for OPERA predictions for the two modes (with and without salts information) to those of the EPI Suite model. RMSEs are 20 °C lower using salts for the training set and 10 °C lower for the test set. However, even without the salts information, the OPERA model MP prediction RMSE is still more than 50 °C lower than EPI Suite model’s RMSE.

**Table 8:** OPERA and EPI Suite MP prediction statistics for chemicals with salts

Dataset	Chemicals with salts	RMSE OPERA (°C)		RMSE EPI Suite (°C)
		No salts	With salts	
Training set	117	102.18	81.56	154.78
Test set	38	98.73	88.68	154.42

Table 7 shows that predicting the MP for chemicals with salts is not easy because RMSE values are higher than the global RMSE values for the two modes. The OPERA MP model is robust, with stable performance across training, fivefold CV, and test steps (RMSE of about 50 °C), but the model can be further improved by adding more experimental data from the literature. A recent work by Tetko et al. [143] reports an RMSE of 32 °C for a model built on a dataset of 300,000 chemicals. However, this accuracy required 700,000 descriptors and expensive computational time, a tradeoff in model complexity. This large data set can be filtered down and added to the used PHYSPROP MP data to improve OPERA MP model accuracy and AD coverage and still comply with OECD principles.

### OPERA model improvements

Since the initial development of the OPERA models using only the curated PHYSPROP dataset, additional changes have been made to the datasets before rebuilding the models. The experimental data have been updated by removing outliers (with extreme values) and adding data from other sources (for properties such as logP, BCF, and RB) [18, 40, 112, 130]. The models have also been refined and refitted, and the code has been optimized for speed and consistency. A new model predicting liquid chromatography retention time (RT) at a 95% confidence window of ± 4.5 min was developed as described in McEachran et al. [144] and also added to OPERA. The EPA is engaged in research linking high resolution mass spectrometry data with high-throughput environmental monitoring [145] and is using the Dashboard to support the identification of “known unknowns” that benefits from OPERA models [146]. Additional parameters to assist in the identification of chemicals based on molecular formula search hit lists is required, and predicted RTs can be a valuable parameter for this purpose. The OPERA RT model has already been used in a non-targeted screening analysis of drinking water conducted at the EPA [147]. OPERA logP, MP, VP and WS models were used in a recent environmental fate assessment study at the EPA showing good performance and room for improvement as well [122]. Additionally, OPERA models were used in a recent study to assess alternative risk assessment methods and inform the development of fit-for-purpose in vitro assays [148].

The current version of OPERA (version 1.5) on Github was used to predict properties for the Dashboard release in August 2017. Since that period, we have continued to collect new data for RT, HL, logP, VP, and WS, and these data will be added to the existing training sets to refine the OPERA models [149]. With these additional data, further analysis including but not limited to Williams graphs for outlier detection and structure–activity landscapes for activity cliff detection will be carried out prior to modeling. The use of other fitting methods and validation techniques will be investigated and the resulting best performing models will be implemented as additional predictive options in OPERA. New environmentally relevant endpoints will also continue to be added to OPERA as data become available. Web services providing real-time prediction capabilities based on SMILES-based structural inputs are presently in development, and the ability to draw a chemical structure in an entry web page as an input to all OPERA models is planned.

## Conclusions

The OPERA suite of prediction models was initially developed based on curated data from the public version of the PHYSPROP database. The ultimate goal of this project is to support regulatory decisions. Therefore, the modeling procedure used to develop OPERA is based on the five OECD principles: well-defined physicochemical and environmental fate endpoints; predictive yet unambiguous algorithms used to fit the models; predictive ability assessed using different conventional methods; a thoroughly defined AD; and mechanistic interpretation of the used descriptors researched and provided in QMRFs validated by the JRC (see Additional file 1: S1). The open-source OPERA code, data, executables, and QMRFs all are freely available under the Massachusetts Institute of Technology (MIT) open license.

OPERA models were used to predict properties for chemical structures contained within the DSSTox database, and the prediction results and reliability assessment reports are available on the EPA’s CompTox Chemistry Dashboard. OPERA data and prediction models will be continuously updated and will follow the regular releases of the Dashboard. Next versions will include more data from different sources, optimized code for speed and accuracy, and new features including, but not limited to, embedding the QSAR-ready structure generation workflow in the dashboard to allow real-time calculation of properties for new structures. Feedback from the users of the Dashboard regarding the models’ performance and assessment provides useful input and is taken into account in the development of iterative versions.

## Additional files

**Additional file 1: S1.** Training and test sets of the models with the corresponding JRC validated QMRFs.

**Additional file 2: S2.** OPERA command line help file.

**Additional file 3: S3.** An example Excel table downloaded from the Chemistry Dashboard with predicted OPERA values.

## Abbreviations

AD: applicability domain
AOH: atmospheric hydroxylation rate
BA: balanced accuracy
BCF: bioconcentration factor
BioHL: biodegradability half-life
BP: boiling point
CASRN: Chemical Abstracts Service Registry Number
CV: cross validation
DSSTox: Distributed Structure-Searchable Toxicity
DTXSID: DSSTox database substance identifier
EPA: U.S. Environmental Protection Agency
FN: false negative
FP: false positive
GA: genetic algorithm
HL: Henry’s law constant
HTS: high-throughput screening
InChI: International Chemical Identifier
IVIVE: in vitro to in vivo extrapolation
JRC: Joint Research Center
KM: fish biotransformation half-life
KNIME: Konstanz Information Miner
kNN: k-nearest neighbor
KOA: octanol–air partition coefficient
KOC: soil adsorption coefficient
logP: octanol–water partition coefficient
MDS: multidimensional scaling
MP: melting point
NCCT: National Center for Computational Toxicology
NHANES: National Health and Nutrition Examination Survey
NICEATM: National Toxicology Program Interagency Center for the Evaluation of Alternative Toxicological Methods
OECD: Organization for Economic Cooperation and Development
OPERA: OPEn structure–activity Relationship App
PBDE: polybrominated diphenyl ether
Q^2^: predictive squared correlation coefficient
QMRF: QSAR model reporting format
QSAR: quantitative structure–activity relationship
QSPR: quantitative structure–property relationship
R^2^: coefficient of determination
RB: readily biodegradable
RMSE: root mean square error
RMSEP: root mean square error in prediction
RT: retention time
SI: supporting information
SMILES: Simplified Molecular Input Line Entry Specification
Sn: sensitivity, the true positive rate
Sp: specificity, the true negative rate
TN: true negative
TP: true positive
VP: vapor pressure
WS: water solubility
