# Supplementary material for: OPERA models for predicting physicochemical properties and environmental fate endpoints
Source: J Cheminform. 2018 Mar 8;10:10. doi: 10.1186/s13321-018-0263-1 (PMC5843579; doi:10.1186/s13321-018-0263-1)
Supplement: Supplementary file 1 — Additional file 1: S1. Training and test sets of the models with the corresponding JRC validated QMRFs. [file 13321_2018_263_MOESM1_ESM.zip › OPERA_BioHL/Q17-23b-0022_BioHL.pdf]

| | |
|---|---|
| QMRF | *QMRF identifier (JRC Inventory):* **Q17-23b-0022** |
| | *QMRF Title:* OPERA-**model for Biodegradation half-life** |
| | *Printing Date:* **Oct 17, 2017** |
| | |

## 1.QSAR identifier

### 1.1.QSAR identifier (title):

OPERA-model for Biodegradation
half-life

### 1.2.Other related models:

No related models

### 1.3.Software coding the model:

OPERA V1.5

OPERA (OPEn (quantitative) structure-activity Relationship Application) is a standalone free and open source command line application. It provides a suite of QSAR models to predict physicochemical properties and environmental fate of organic chemicals based on PaDEL descriptors. It is available for download in Matlab, C and C++ languages from github under MIT license.

Kamel Mansouri (mansourikamel@gmail.com)

https://github.com/kmansouri/OPERA.git

PaDEL descriptors V2.21

Open source software to calculate molecular descriptors and fingerprints.

Chun Wei Yap (phayapc@nus.edu.sg)

http://padel.nus.edu.sg/software/padeldescriptor

MATLAB V8.2

MATrix LABoratory is a multi-paradigm numerical computing environment and fourth-generation programming language

http://www.mathworks.com/company/aboutus/contact_us/?s_tid=gn_cntus

http://www.mathworks.com/products/matlab/

## 2.General information

### 2.1.Date of QMRF:

1 November 2016

### 2.2.QMRF author(s) and contact details:


[1]Kamel Mansouri, ORISE research fellow at National Center for Computational Toxicology (NCCT), U.S. Environmental Protection Agency, mansourikamel@gmail.com

[2]Antony Williams, National Center for Computational Toxicology (NCCT), U.S. Environmental Protection Agency, Williams.Antony@epa.gov


### 2.3.Date of QMRF update(s):

### 2.4.QMRF update(s):

### 2.5.Model developer(s) and contact details:

Kamel Mansouri, ORISE research fellow at National Center for Computational Toxicology (NCCT), U.S. Environmental Protection Agency, mansourikamel@gmail.com

**2.6.Date of model development and/or publication:**

2016

**2.7.Reference(s) to main scientific papers and/or software package:**

[1]An automated curation procedure for addressing chemical errors and inconsistencies in public datasets used in QSAR modeling. 2016. Kamel Mansouri, Chris M. Grulke, Ann M. Richard, Richard S. Judson and Antony J. Williams. SAR & QSAR in Environ. Res; Vol. 27 , Iss. 11,2016. doi: 10.1080/1062936X.2016.1253611.

[2]OPERA: A free and open source QSAR tool for physicochemical properties and environmental fate predictions. Kamel Mansouri, Chris Grulke, Richard Judson, Antony Williams, Journal of Cheminformatics (2017)

[3]PaDEL-descriptor: an open source software to calculate molecular descriptors and fingerprints. Chun Wei Yap. (2011). J. Comput. Chem., 32: 1466–1474. doi:10.1002/jcc.21707 http://onlinelibrary.wiley.com/doi/10.1002/jcc.21707/abstract

[4]A KNIME workflow for chemical structures curation and standardization in QSAR modeling. Kamel Mansouri, Sherif Farag, Jayaram Kancherla, Regina Politi, Eugene Muratov, Denis Fourches, Nikolai Nikolov, Eva Bay Wedebay, Christopher Grulke, Ann Richard, Richard Judson, Alexander Tropsha. (in preparation)

[5]The influence of data curation on QSAR Modeling – examining issues of quality versus quantity of data (SOT). Williams, A., K. Mansouri, A. Richard, AND C. Grulke. Presented at Society of Toxicology, New Orleans, LA, March 13 - 17, 2016. https://cfpub.epa.gov/si/si_public_record_report.cfm?dirEntryId=311418

[6]An Online Prediction Platform to Support the Environmental Sciences (American Chemical Society). Richard, A., C. Grulke, K. Mansouri, R. Judson, AND A. Williams. Presented at ACS Spring Meeting, San Diego, CA, March 13 - 17, 2016. https://cfpub.epa.gov/si/si_public_record_Report.cfm?dirEntryId=311655

[7]The importance of data curation on QSAR Modeling: PHYSPROP open data as a case study. Kamel Mansouri, Christopher Grulke Ann Richard Richard Judson Antony Williams. Presented at QSAR2016 14 June 2016, Miami, FL http://www.qsar2016.com/program

[8]Mansouri K. (2017) OPERA: A QSAR tool for physicochemical properties and environmental fate predictions. doi: 10.6084/m9.figshare.4836428 https://figshare.com/articles/OPERA_A_QSAR_tool_for_physicochemical_properties_and_environmental_fate_predictions/4836428

**2.8.Availability of information about the model:**

Non-proprietary suite of QSAR models freely available as a command line standalone application (OPERA: OPEn saR App) from github under MIT license: https://github.com/kmansouri/OPERA.git. Its predictions for the full DSSTox 720k chemicals are published on the EPA CompTox Chemistry Dashboard ( https://comptox.epa.gov/dashboard). Training and validation sets are available for visualization on the dashboard and as SDF files provided in supporting information Section 9.3 and from the paper [ref 1-2, Section 2.7]. ( ftp://newftp.epa.gov/COMPTOX/Sustainable_Chemistry_Data/Chemistry_Dashboard/PHYSPROP_Analysis)

### 2.9.Availability of another QMRF for exactly the same model:

Not to date

## 3.Defining the endpoint - OECD Principle 1

### 3.1.Species:

Not applicable

### 3.2.Endpoint:

2.Environmental fate parameters 2.3.b.Persistence: Biodegradation. Biodegradation time frame (primary, ultimate degradation)

### 3.3.Comment on endpoint:

Biodegradation half-lifefor compounds containing only carbon and hydrogen (i.e. hydrocarbons).

### 3.4.Endpoint units:

Log days

### 3.5.Dependent variable:

Log HalfLife

### 3.6.Experimental protocol:

The experimental data were downloaded from the EPI Suite data webpage (http://esc.syrres.com/interkow/EpiSuiteData.htm). These data are from PHYSPROP (The Physical Properties Database) which is a collection of a wide variety of sources built by Syracuse Research Corporation (SRC). Experimental protocols of the different parts of data can be traced back to the original referenced literature from the database.

### 3.7.Endpoint data quality and variability:

The original data collected from the PHYSPROP database (175 chemicals) have undergone a series of processes to curate the chemical structures and remove duplicates, obvious outliers and erroneous entries. This procedure also included a consistency check to ensure only good quality data is used for the development of the QSAR model (151 chemicals).

Then, QSAR-ready structures were generated by standardizing all chemical structures and removing duplicates, inorganic and metallo-organic chemicals (150 chemicals). The descriptions of KNIME workflows that were developed for the purpose of the cleaning and standardization of the data are available in the papers [ref 1 and ref 4 Section 2.7]. The curated outlier-free experimental data (150 chemicals) was divided into training and validation sets before the machine learning and modeling steps.

## 4.Defining the algorithm - OECD Principle 2

### 4.1.Type of model:

QSAR/QSPR model derived derived from weighted nearest neighbors algorithm (kNN) based on PaDEL descriptors [ref2 Sect 1.3].

### 4.2.Explicit algorithm:

Distance weighted k-nearest neighbors (kNN). k=5.

This is a refinement of the classical k-NN classification algorithm where the contribution of each of the k neighbors is weighted according to their distance to the query point, giving greater weight to closer neighbors.The used distance is the Euclidean distance. kNN is an unambiguous algorithm that fulfills the transparency requirements of OECD principle 2 with an optimal compromise between model complexity and performance.

**4.3.Descriptors in the model:**

[1]BCUTp-1l, Unitless, Polarizability weighted BCUTS. Pearlman, R.S. and Smith, K.M., Metric Validation and the Receptor-Relevant Subspace Concept, J. Chem. Inf. Comput. Sci., 1999, 39:28-35; Burden, F.R., Molecular identification number for substructure searches , J. Chem. Inf. Comput. Sci., 1989, 29:225-227; Burden, F.R., Chemically Intuitive Molecular Index, Quant. Struct .-Act. Relat., 1997, 16:309-314;

[2]IC2, Unitless, Information content index (neighborhood symmetry of 2-order). Todeschini, R. and Consonni, V. (2009). Molecular descriptors for chemoinformatics, (Weinheim: Wiley VCH) pg 408-411.

[3]nF6Ring, Unitless, Ring count: Number of 6-membered fused rings.

[4]ATSC1p, Unitless, Centered Broto-Moreau autocorrelation - lag 1 / weighted by polarizabilities. Todeschini, R. and Consonni, V. (2009). Molecular descriptors for chemoinformatics, (Weinheim: Wiley VCH) pg 27-37

[5]nT6Ring, Unitless, Number of 6-membered rings (includes counts from fused rings)

[6]VP-6, Unitless, Chi path: Valence path, order 6. Kier, L. B., and Hall, L. H. (1976). Molecular connectivity in chemistry and drug research, (New York: Academic Press).

**4.4.Descriptor selection:**

PaDEL software was used to calculate 1440 molecular descriptors. A first filter was applied in order to remove descriptors with missing values, constant and near constant (standard deviation of 0.25 as a threshold) and highly correlated descriptors (96% as a threshold). The remaining 650 descriptors were used in a feature selection procedure to select a minimum number of variables encoding the most relevant structural information to the modeled endpoint. This step consisted of coupling Genetic Algorithms (GA) with the weighted kNN algorithm and was applied in 5 fold cross validation on the training set (112 chemicals). This procedure was run for 200 consecutive independent runs maximizing $Q^2$ in cross-validation and minimizing the number of descriptors. The number of k neighbors is optimized within the range of 3 to 7. The descriptors were then ranked based on their frequency of selection during the GA runs. The best model showed an optimal compromise between the simplicity (minimum number of descriptors) and performance ($Q^2$ in cross-validation) to ensure transparency and facilitate the mechanistic interpretation as required by OECD principles 2 and 5. More details in paper [ref2 Section 2.7].

**4.5.Algorithm and descriptor generation:**

PaDEL descriptors were calculated based on two-dimensional (2D) chemical structures generated by the Indigo cheminformatics suite of tools implemented in KNIME. 2D descriptors were selected over 3D to avoid complicated and usually irreproducible

geometrical optimizations. The calculated descriptors fall into different groups such as constitutional indices, ring descriptors, topological indices, 2D matrix based descriptors, functional group counts and atom counts. Details and references provided in Section 4.3.

**4.6.Software name and version for descriptor generation:**

PaDEL-Descriptors V2.21

An open source software to calculate molecular descriptors and fingerprints.

Chun Wei Yap (phayapc@nus.edu.sg)

http://padel.nus.edu.sg/software/padeldescriptor

**4.7.Chemicals/Descriptors ratio:**

112 chemicals (trainingset)/6 descriptors= 18.66

## 5.Defining the applicability domain - OECD Principle 3

**5.1.Description of the applicability domain of the model:**

The model is applicable to heterogeneous organic chemicals. In the implementation of the model several pieces of information are given to help the user in evaluating the reliability of a prediction. The chemical structure is first assessed to see if it is falling within the Applicability Domain of the model or not. Then the accuracy of the predicted value is reported based on the similarity of the query chemical to its neighboring chemicals in the training set of the model.

This fullfills the requirements of the 3rd OECD principle by defining the limitations in terms of the types of chemical structures, physicochemical properties and mechanisms of action for which the model can generate reliable predictions.

**5.2.Method used to assess the applicability domain:**

The applicability domain of the model is assessed in two independent levels using two different distance-based methods. First, a global applicability domain is determined by means of the leverage approach that checks whether the query structure falls within the multidimensional chemical space of the whole training set.

The leverage of a query chemical is proportional to its Mahalanobis distance measure from the centroid of the training set. The leverages of a given dataset are obtained from the diagonal values of the hat matrix.

This approach is associated with a threshold leverage that corresponds to 3*p/n where p is the number of model variables while n is the number of training compounds. A query chemical with leverage higher than the threshold is considered outside the AD and can be associated with unreliable prediction.

The leverage approach has specific limitations, in particular with respects to gaps within the descriptor space of the model or at the boundaries of the training set. To obviate such limitations, a second tier of applicability domain assessement was added. This comprised a local approach which only investigated the vicinity of the query chemical. This local approach provides a continuous index ranging from 0 to 1 which is different from the first approach which only provides

Boolean answers (yes/no). This local AD-index is relative to the similarity of the query chemical to its 5 nearest neighbors in the p dimensional space of the model. The higher this index, the more the prediction is likely to be reliable.

**5.3.Software name and version for applicability domain assessment:**

Implemented in OPERA V1.5

An implementation of a local similarity index and the leverage approach based on the work of Sahigara, F.; Mansouri, K.; Ballabio, D.; Mauri, A.; Consonni, V.; Todeschini, R. Comparison of Different Approaches to Define the Applicability Domain of QSAR Models. Molecules 2012, 17, 4791-4810.

Kamel Mansouri (mansourikamel@gmail.com);

https://github.com/kmansouri/OPERA.git

**5.4.Limits of applicability:**

These two AD methods described in Section 5.2 are complementary and can be interpreted in the following way:

- If a chemical is considered outside the global AD with a low local AD-index, the prediction can be unreliable
- If a chemical is considered outside the global AD but the local AD-index is average or relatively high, this means the query chemical is on the boundaries of the training set but has quite similar neighbors. The prediction can be trusted.
- If a chemical is considered inside the global AD but the local AD-index is average or relatively low, this means the query chemical fell in a "gap" of the chemical space of the model but still within the boudaries of the training set and surrounded with training chemicals. The prediction should be considered with caution.
- If a chemical is considered inside the global AD with a high local AD-index, the prediction should be considered reliable.

Even though the applicability domain is necessary to set the limits of the interpolation space of the model, it doesn't necessarily inform about the quality of the prediction especially in the empty spaces and around the edges of the descriptor space. In order to overcome this limitation and help the user decide about the reliability of a prediction, we added a confidence level index ranging from 0 to 1 relative to the accuracy of prediction of the 5 nearest neighbors to the query chemical. The higher this index, the more the prediction is likely to be reliable.

## 6.Internal validation - OECD Principle 4

**6.1.Availability of the training set:**

Yes

**6.2.Available information for the training set:**

Internal ID; CAS checksum; name validity; preferred name; IUPAC name; Original SMILES; QSAR-ready canonical smiles; InChI; Salt information; DSSTox GSID; Experimental reference; Consistency flag

CAS RN: Yes

Chemical Name: Yes

Smiles: Yes

Formula: No

INChI: Yes

MOL file: Yes

**6.3.Data for each descriptor variable for the training set:**

All

**6.4.Data for the dependent variable for the training set:**

All

**6.5.Other information about the training set:**

The training set consists of 112 chemicals. The structures are randomly selected to represent 75% of the available data keeping a similar normal distrubution of LogHalfLife vlaues in both training and test sets using the Venetian blinds method. The values are ranging from ~0.3 to ~3. A plot of the distribution of LogHalffLife values is provided in the supporting information Section 9.3.

**6.6.Pre-processing of data before modelling:**

No preprocessing of the values.

**6.7.Statistics for goodness-of-fit:**

Performance in training:

$R^2$=0.88

RMSE=0.26

**6.8.Robustness - Statistics obtained by leave-one-out cross-validation:**

**6.9.Robustness - Statistics obtained by leave-many-out cross-validation:**

Performance in 5-fold cross-validation:

$Q^2$=0.89

RMSE=0.25

A plot of the experimental versus predicted values for the training set is provided in supporting information Section 9.3.

**6.10.Robustness - Statistics obtained by Y-scrambling:**

**6.11.Robustness - Statistics obtained by bootstrap:**

**6.12.Robustness - Statistics obtained by other methods:**

## 7.External validation - OECD Principle 4

**7.1.Availability of the external validation set:**

Yes

**7.2.Available information for the external validation set:**

Internal ID; CAS checksum; name validity; preferred name; IUPAC name; Original SMILES; QSAR-ready canonical smiles; InChI; Salt information; DSSTox GSID; Experimental reference; Consistency flag

CAS RN: Yes

Chemical Name: Yes

Smiles: Yes

Formula: No

INChI: Yes

MOL file: Yes

**7.3.Data for each descriptor variable for the external validation set:**

All

**7.4.Data for the dependent variable for the external validation set:**

All

**7.5.Other information about the external validation set:**

The validation set consists of 38 chemicals.

The values are ranging from ~0.3 to ~2.5.

**7.6.Experimental design of test set:**

The structures are randomly selected to represent 25% of the available data keeping a similar normal distrubution of LogHalfLife vlaues in both training and test sets using the Venetian blinds method. A plot of the distribution of LogHalfLife values is provided in the supporting information Section 9.3.

**7.7.Predictivity - Statistics obtained by external validation:**

Performance in test:

$R^2$=0.75

RMSE=0.38

**7.8.Predictivity - Assessment of the external validation set:**

The validation set consisting of 38 chemicals which is equivalent to a third (1/3) of the training set is sufficient for the evaluation of the predictivity of the model and a good representation of the chemical space as shown in the multi-dimensional scaling plot provided in supporting information Section 9.3. A plot of the experimental versus predicted values for the validation set is provided in supporting information Section 9.3.

**7.9.Comments on the external validation of the model:**

The choice of proportions between the training set and the validation set as well as the splitting method helped in accurately evaluating the model and covering most of the training set chemical space. This goal was accomplished without the need to do a structural sampling that usually shows over-optimistic evaluation of the predictivity or a complete random selection that risks biasing the evaluation towards a certain region of the chemical space.

## 8.Providing a mechanistic interpretation - OECD Principle 5

**8.1.Mechanistic basis of the model:**

The model descriptors were selected statistically but they can also be mechanistically interpreted.

Mansouri et al. 2013 provided a mechanistic interpretation of molecular descriptors related to biodegradability of organic chemicals [ref 1 Section 9.2]. They confirmed literature sources stating that cyclicity

decreases biodegradability [ref 2 Section 9.2]. Here we used two descriptors encoding ring information (nF6Ring and nT6Ring).

It was demonstrated that water soluble molecules tend to be more easily biodegradable than insoluble ones [ref 3 Section 9.2]. Here we used two molecular descriptors that encode information about polarizability (BCUTp-11 and ATSC1p) which is correlated with water solubility.

IC2 and VP-6 inform about the branching of the chemicals which was reported in the literature to have a negative influence on the biodegradability of organic chemicals [ref 1 Section 9.2].

### 8.2.A priori or a posteriori mechanistic interpretation:

A posteriori mechanistic interpretation.

### 8.3.Other information about the mechanistic interpretation:

For more details and full reference, see references in Section 4.3 and Section 9.2.

## 9.Miscellaneous information

### 9.1.Comments:

This QSAR model for LogHalfLife prediction is part of the NCCT_Models Suite that is a free and open-source standalone application for the prediction of physicochemical properties and environmental fate of chemicals. This application is available in the Supporting information Section 9.3 of this report and in the paper ref 2 Section 2.7. The detailed results of this suite of models applied on more than 700k DSSTox chemicals are available on the iCSS chemistry dashboard (https://comptox.epa.gov/dashboard).

This current version of the model is mainly based on curated and standardized data collected from the Physprop database. All NCCT_Models are designed to fulfil the requirement of the 5 OECD principles to ensure transparency and reproducibility of the results. In order to predict new chemicals, the models only require 2D chemical structures that are used to calculate molecular descriptors by PaDEL 2.21 software. Then a simple weighted kNN algorithm is used to make the prediction based on the observed values of the k closest molecules. All models showed high robustness and statistics stability between training, 5-fold cross-validation and the external validation set.

Considering the full applicability domain of the 150 chemicals with available data and the same models parameters described earlier, the calibration statistics would be an $R^2$ of 0.84 and an RMSE of 0.29.

### 9.2.Bibliography:

[1]Boethling RS (1996) Designing Biodegradable Chemicals. In Designing Safer Chemicals; DeVito SC, Garrett R L, Eds.; American Chemical Society: Washington, DC, 640, 156171.

[2]Mansouri K, Ringsted T, Ballabio D,* Todeschini Rand Consonni V (2013) Quantitative StructureActivity Relationship Models for Ready Biodegradability of Chemicals. J. Chem. Inf. Mode 53, 867878
[3]Klopman G, Balthasar DM, Rosenkranz HS (1993) Application of the computer-automated structure evaluation (CASE) program to the study of structure-biodegradation relationships of miscellaneous chemicals. Environ. Toxicol. Chem. 12, 231240.

**9.3.Supporting information:**

## 10.Summary (JRC QSAR Model Database)

**10.1.QMRF number:**

Q17-23b-0022

**10.2.Publication date:**

2017-09-21

**10.3.Keywords:**

Biodegradation half-life;PaDEL;OPERA;

**10.4.Comments:**

To be entered by JRC
