# Supplementary material for: OPERA models for predicting physicochemical properties and environmental fate endpoints
Source: J Cheminform. 2018 Mar 8;10:10. doi: 10.1186/s13321-018-0263-1 (PMC5843579; doi:10.1186/s13321-018-0263-1)
Supplement: Supplementary file 1 — Additional file 1: S1. Training and test sets of the models with the corresponding JRC validated QMRFs. [file 13321_2018_263_MOESM1_ESM.zip › OPERA_KOC/Q17-26-0017_Log KOC.pdf]

QMRF	*QMRF identifier (JRC Inventory):*Q17-26-0017
	*QMRF Title:*OPERA-model for organic carbon-sorption coefficient
	*Printing Date:*Oct 17, 2017

1.QSAR identifier

1.1.QSAR identifier (title):

OPERA-model for organic carbon-sorption coefficient

1.2.Other related models:

No related models

1.3.Software coding the model:

OPERA V1.5

OPERA (OPEn (quantitative) structure-activity Relationship Application) is a standalone free and open source command line application. It provides a suite of QSAR models to predict physicochemical properties and environmental fate of organic chemicals based on PaDEL descriptors. It is available for download in Matlab, C and C++ languages from github under MIT license.

Kamel Mansouri (mansourikamel@gmail.com)

https://github.com/kmansouri/OPERA.git

PaDEL descriptors V2.21

Open source software to calculate molecular descriptors and fingerprints.

Chun Wei Yap (phayapc@nus.edu.sg)

http://padel.nus.edu.sg/software/padeldescriptor

MATLAB V8.2

MATrix LABoratory is a multi-paradigm numerical computing environment and fourth-generation programming language

http://www.mathworks.com/company/aboutus/contact_us/?s_tid=gn_cntus

http://www.mathworks.com/products/matlab/

2.General information

2.1.Date of QMRF:

1 November 2016

2.2.QMRF author(s) and contact details:

[1]Kamel Mansouri, ORISE research fellow at National Center for Computational Toxicology (NCCT), U.S. Environmental Protection Agency, mansourikamel@gmail.com

[2]Antony Williams, National Center for Computational Toxicology (NCCT), U.S. Environmental Protection Agency, Williams.Antony@epa.gov

2.3.Date of QMRF update(s):

2.4.QMRF update(s):

2.5.Model developer(s) and contact details:

Kamel Mansouri, ORISE research fellow at National Center for Computational Toxicology (NCCT), U.S. Environmental Protection Agency, mansourikamel@gmail.com

2.6.Date of model development and/or publication:

2016

2.7.Reference(s) to main scientific papers and/or software package:

[1]An automated curation procedure for addressing chemical errors and inconsistencies in public datasets used in QSAR modeling. 2016. Kamel Mansouri, Chris M. Grulke, Ann M. Richard, Richard S. Judson and Antony J. Williams. SAR & QSAR in Environ. Res; Vol. 27 , Iss. 11,2016. doi: 10.1080/1062936X.2016.1253611.

[2]OPERA: A free and open source QSAR tool for physicochemical properties and environmental fate predictions. Kamel Mansouri, Chris Grulke, Richard Judson, Antony Williams, Journal of Cheminformatics (2017)

[3]PaDEL-descriptor: an open source software to calculate molecular descriptors and fingerprints. Chun Wei Yap. (2011). J. Comput. Chem., 32: 1466–1474. doi:10.1002/jcc.21707 http://onlinelibrary.wiley.com/doi/10.1002/jcc.21707/abstract

[4]A KNIME workflow for chemical structures curation and standardization in QSAR modeling. Kamel Mansouri, Sherif Farag, Jayaram Kancherla, Regina Politi, Eugene Muratov, Denis Fourches, Nikolai Nikolov, Eva Bay Wedebay, Christopher Grulke, Ann Richard, Richard Judson, Alexander Tropsha. (in preparation)

[5]The influence of data curation on QSAR Modeling – examining issues of quality versus quantity of data (SOT). Williams, A., K. Mansouri, A. Richard, AND C. Grulke. Presented at Society of Toxicology, New Orleans, LA, March 13 - 17, 2016. https://cfpub.epa.gov/si/si_public_record_report.cfm?dirEntryId=311418

[6]An Online Prediction Platform to Support the Environmental Sciences (American Chemical Society). Richard, A., C. Grulke, K. Mansouri, R. Judson, AND A. Williams. Presented at ACS Spring Meeting, San Diego, CA, March 13 - 17, 2016. https://cfpub.epa.gov/si/si_public_record_Report.cfm?dirEntryId=311655

[7]The importance of data curation on QSAR Modeling: PHYSPROP open data as a case study. Kamel Mansouri, Christopher Grulke Ann Richard Richard Judson Antony Williams. Presented at QSAR2016 14 June 2016, Miami, FL http://www.qsar2016.com/program

[8]Mansouri K. (2017) OPERA: A QSAR tool for physicochemical properties and environmental fate predictions. doi: 10.6084/m9.figshare.4836428 https://figshare.com/articles/OPERA_A_QSAR_tool_for_physicochemical_properties_and_environmental_fate_predictions/4836428

2.8.Availability of information about the model:

Non-proprietary suite of QSAR models freely available as a command line standalone application (OPERA: OPEn saR App) from github under MIT license: https://github.com/kmansouri/OPERA.git. Its predictions for the full DSSTox 720k chemicals are published on the EPA CompTox Chemistry Dashboard (https://comptox.epa.gov/dashboard). Training and validation sets are available for visualization on the dashboard and as SDF files provided in supporting information Section 9.3 and from the paper [ref 1-2, Section 2.7]. (ftp://newftp.epa.gov/COMPTOX/Sustainable_Chemistry_Data/Chemistry_Dashboard/PHYSPROP_Analysis)

2.9.Availability of another QMRF for exactly the same model:

Not to date

3.Defining the endpoint - OECD Principle 1

3.1.Species:

Not applicable

3.2.Endpoint:

2.Environmental fate parameters 2.6.Partition coefficient. Organic carbon-sorption partition coefficient (organic carbon; Koc)

3.3.Comment on endpoint:

The soil adsorption coefficient is the ratio of the amount of chemical adsorbed per unit weight of organic carbon in the soil or sediment to the concentration of the chemical in solution at equilibrium (in L/Kg).

3.4.Endpoint units:

Log L/Kg

3.5.Dependent variable:

LogKoc

3.6.Experimental protocol:

The experimental data were downloaded from the EPI Suite data webpage (http://esc.syrres.com/interkow/EpiSuiteData.htm). These data are from PHYSPROP (The Physical Properties Database) which is a collection of a wide variety of sources built by Syracuse Research Corporation (SRC). Experimental protocols of the different parts of data can be traced back to the original referenced literature from the database.

3.7.Endpoint data quality and variability:

The original data collected from the PHYSPROP database (788 chemicals) have undergone a series of processes to curate the chemical structures and remove duplicates, obvious outliers and erroneous entries. This procedure also included a consistency check to ensure only good quality data is used for the development of the QSAR model (750 chemicals).

Then, QSAR-ready structures were generated by standardizing all chemical structures and removing duplicates, inorganic and metallo-organic chemicals (735 chemicals). The descriptions of KNIME workflows that were developed for the purpose of the cleaning and standardization of the data are available in the papers [ref 1 and ref 4 Section 2.7].

The curated outlier-free experimental data (729 chemicals) was divided into training and validation sets before the machine learning and modeling steps.

4.Defining the algorithm - OECD Principle 2

4.1.Type of model:

QSAR/QSPR model derived derived from weighted nearest neighbors algorithm (kNN) based on PaDEL descriptors [ref2 Sect 1.3].

4.2. Explicit algorithm:

Distance weighted k-nearest neighbors (kNN). k=5

This is a refinement of the classical k-NN classification algorithm where the contribution of each of the k neighbors is weighted according to their distance to the query point, giving greater weight to closer neighbors.The used distance is the Euclidean distance. kNN is an unambiguous algorithm that fulfills the transparency requirements of OECD principle 2 with an optimal compromise between model complexity and performance.

4.3. Descriptors in the model:

[1]CrippenLogP, Unitless, Estimate of partion coefficients. Wildman, S. A., and Crippen, G. M. (1999). Prediction of Physicochemical Parameters by Atomic Contributions. J Chem Inf Comput Sci 39, 868-873.

[2]nHBAcc, Unitless, Number of hydrogen bond acceptors (using CDK HBondAcceptorCountDescriptor algorithm)

[3]SpDiam_Dzi, Unitless, Barysz matrix: Spectral diameter from Barysz matrix / weighted by first ionization potential. Todeschini, R. and Consonni, V. (2009). Molecular descriptors for chemoinformatics, (Weinheim: Wiley VCH) pg 714-726

[4]VP-1, Unitless, Chi path: Valence path, order 1. Kier, L. B., and Hall, L. H. (1976). Molecular connectivity in chemistry and drug research, (New York: Academic Press).

[5]MPC3, Unitless, Path counts: Molecular path count of order 3. Todeschini, R. and Consonni, V. (2009). Molecular descriptors for chemoinformatics, (Weinheim: Wiley VCH) pg 574-579, pg 395-402

[6]TPC, Unitless, Path counts: Total path count (up to order 10). Todeschini, R. and Consonni, V. (2009). Molecular descriptors for chemoinformatics, (Weinheim: Wiley VCH) pg 574-579, pg 395-402

[7]VABC, Unitless, Van der Waals volume: Van der Waals volume calculated using the method proposed in [Zhao, Yuan H. and Abraham, Michael H. and Zissimos, Andreas M., Fast Calculation of van der Waals Volume as a Sum of Atomic and Bond Contributions and Its Application to Drug Compounds, The Journal of Organic Chemistry, 2003, 68:7368-7373]

[8]SsssN, Unitless, Sum of atom-type E-State: >N-

[9]topoDiameter, Unitless, Topological diameter (maximum atom eccentricity)

[10]AATS1m, Unitless, Average Broto-Moreau autocorrelation - lag 1 / weighted by mass. Todeschini, R. and Consonni, V. (2009). Molecular descriptors for chemoinformatics, (Weinheim: Wiley VCH) pg 27-37

[11]MPC6, Unitless, Path counts: Molecular path count of order 6. Todeschini, R. and Consonni, V. (2009). Molecular descriptors for chemoinformatics, (Weinheim: Wiley VCH) pg 574-579, pg 395-402

[12]ATS1v, Unitless, Broto-Moreau autocorrelation - lag 1 / weighted by van der Waals volumes. Todeschini, R. and Consonni, V. (2009). Molecular descriptors for chemoinformatics, (Weinheim: Wiley VCH) pg 27-37

4.4. Descriptor selection:

PaDEL software was used to calculate 1440 molecular descriptors. A first filter was applied in order to remove descriptors with missing values, constant and near constant (standard deviation of 0.25 as a threshold) and highly correlated descriptors (96% as a threshold). The remaining 845 descriptors were used in a feature selection procedure to select a minimum number of variables encoding the

most relevant structural information to the modeled endpoint. This step consisted of coupling Genetic Algorithms (GA) with the weighted kNN algorithm and was applied in 5 fold cross validation on the training set (545 chemicals). This procedure was run for 200 consecutive independent runs maximizing Q^2 in cross-validation and minimizing the number of descriptors. The number of k neighbors is optimized within the range of 3 to 7. The descriptors were then ranked based on their frequency of selection during the GA runs. The best model showed an optimal compromise between the simplicity (minimum number of descriptors) and performance (Q^2 in cross-validation) to ensure transparency and facilitate the mechanistic interpretation as required by OECD principles 2 and 5. More details in paper [ref2 Section 2.7].

4.5.Algorithm and descriptor generation:

PaDEL descriptors were calculated based on two-dimensional (2D) chemical structures generated by the Indigo cheminformatics suite of tools implemented in KNIME. 2D descriptors were selected over 3D to avoid complicated and usually irreproducible geometrical optimizations. The calculated descriptors fall into different groups such as constitutional indices, ring descriptors, topological indices, 2D matrix based descriptors, functional group counts and atom counts. Details and references provided in Section 4.3.

4.6.Software name and version for descriptor generation:

PaDEL-Descriptors V2.21
An open source software to calculate molecular descriptors and fingerprints.
Chun Wei Yap (phayapc@nus.edu.sg)
http://padel.nus.edu.sg/software/padeldescriptor

4.7.Chemicals/Descriptors ratio:

405 chemicals (trainingset)/12 descriptors= 33.75

5.Defining the applicability domain - OECD Principle 3

5.1.Description of the applicability domain of the model:

The model is applicable to heterogeneous organic chemicals. In the implementation of the model several pieces of information are given to help the user in evaluating the reliability of a prediction. The chemical structure is first assessed to see if it is falling within the Applicability Domain of the model or not. Then the accuracy of the predicted value is reported based on the similarity of the query chemical to its neighboring chemicals in the training set of the model.

This fullfills the requirements of the 3rd OECD principle by defining the limitations in terms of the types of chemical structures, physicochemical properties and mechanisms of action for which the model can generate reliable predictions.

5.2.Method used to assess the applicability domain:

The applicability domain of the model is assessed in two independent levels using two different distance-based methods. First, a global applicability domain is determined by means of the leverage approach

that checks whether the query structure falls within the multidimensional chemical space of the whole training set.

The leverage of a query chemical is proportional to its Mahalanobis distance measure from the centroid of the training set. The leverages of a given dataset are obtained from the diagonal values of the hat matrix. This approach is associated with a threshold leverage that corresponds to 3*p/n where p is the number of model variables while n is the number of training compounds. A query chemical with leverage higher than the threshold is considered outside the AD and can be associated with unreliable prediction.

The leverage approach has specific limitations, in particular with respects to gaps within the descriptor space of the model or at the boundaries of the training set. To obviate such limitations, a second tier of applicability domain assessement was added. This comprised a local approach which only investigated the vicinity of the query chemical. This local approach provides a continuous index ranging from 0 to 1 which is different from the first approach which only provides Boolean answers (yes/no). This local AD-index is relative to the similarity of the query chemical to its 5 nearest neighbors in the p dimensional space of the model. The higher this index, the more the prediction is likely to be reliable.

5.3.Software name and version for applicability domain assessment:

Implemented in OPERA V1.5

An implementation of a local similarity index and the leverage approach based on the work of Sahigara, F.; Mansouri, K.; Ballabio, D.; Mauri, A.; Consonni, V.; Todeschini, R. Comparison of Different Approaches to Define the Applicability Domain of QSAR Models. Molecules 2012, 17, 4791-4810.

Kamel Mansouri (mansourikamel@gmail.com);

https://github.com/kmansouri/OPERA.git

5.4.Limits of applicability:

These two AD methods described in Section 5.2 are complementary and can be interpreted in the following way:

- If a chemical is considered outside the global AD with a low local AD-index, the prediction can be unreliable
- If a chemical is considered outside the global AD but the local AD-index is average or relatively high, this means the query chemical is on the boundaries of the training set but has quite similar neighbors. The prediction can be trusted.
- If a chemical is considered inside the global AD but the local AD-index is average or relatively low, this means the query chemical fell in a "gap" of the chemical space of the model but still within the boudaries of the training set and surrounded with training chemicals. The prediction should be considered with caution.
- If a chemical is considered inside the global AD with a high local AD-index, the prediction should be considered reliable.

Even though the applicability domain is necessary to set the

limits of the interpolation space of the model, it doesn't necessarily inform about the quality of the prediction especially in the empty spaces and around the edges of the descriptor space. In order to overcome this limitation and help the user decide about the reliability of a prediction, we added a confidence level index ranging from 0 to 1 relative to the accuracy of prediction of the 5 nearest neighbors to the query chemical. The higher this index, the more the prediction is likely to be reliable.

6.Internal validation - OECD Principle 4

6.1.Availability of the training set:

Yes

6.2.Available information for the training set:

Internal ID; CAS checksum; name validity; preferred name; IUPAC name; Original SMILES; QSAR-ready canonical smiles; InChI; Salt information; DSSTox GSID; Experimental reference; Consistency flag

CAS RN: Yes

Chemical Name: Yes

Smiles: Yes

Formula: No

INChI: Yes

MOL file: Yes

6.3.Data for each descriptor variable for the training set:

All

6.4.Data for the dependent variable for the training set:

All

6.5.Other information about the training set:

The training set consists of 545 chemicals. The structures are randomly selected to represent 75% of the available data keeping a similar normal distrubution of LogKoc vlaues in both training and test sets using the Venetian blinds method. The values are ranging from 0 to 6.5. A plot of the distribution of LogKoc values is provided in the supporting information Section 9.3.

6.6.Pre-processing of data before modelling:

No preprocessing of the values.

6.7.Statistics for goodness-of-fit:

Performance in training:

R^2=0.81

RMSE=0.54

6.8.Robustness - Statistics obtained by leave-one-out cross-validation:

6.9.Robustness - Statistics obtained by leave-many-out cross-validation:

Performance in 5-fold cross-validation:

Q^2=0.81

RMSE=0.55

A plot of the experimental versus predicted values for the training set is provided in supporting information Section 9.3.

6.10.Robustness - Statistics obtained by Y-scrambling:

6.11.Robustness - Statistics obtained by bootstrap:

6.12.Robustness - Statistics obtained by other methods:

7.External validation - OECD Principle 4

7.1.Availability of the external validation set:

Yes

7.2.Available information for the external validation set:

Internal ID; CAS checksum; name validity; preferred name; IUPAC name; Original SMILES; QSAR-ready canonical smiles; InChI; Salt information; DSSTox GSID; Experimental reference; Consistency flag

CAS RN: Yes

Chemical Name: Yes

Smiles: Yes

Formula: No

INChI: Yes

MOL file: Yes

7.3.Data for each descriptor variable for the external validation set:

All

7.4.Data for the dependent variable for the external validation set:

All

7.5.Other information about the external validation set:

The validation set consists of 184 chemicals.

The values are ranging from ~-0.3 to ~6.

7.6.Experimental design of test set:

The structures are randomly selected to represent 25% of the available data keeping a similar normal distrubution of LogKoc vlaues in both training and test sets using the Venetian blinds method. A plot of the distribution of LogKoc values is provided in the supporting information Section 9.3.

7.7.Predictivity - Statistics obtained by external validation:

Performance in test:

R^2=0.71

RMSE=0.61

7.8.Predictivity - Assessment of the external validation set:

The validation set consisting of 184 chemicals which is equivalent to a third (1/3) of the training set is sufficient for the evaluation of the predictivity of the model and a good representation of the chemical space as shown in the multi-dimensional scaling plot provided in supporting information Section 9.3. A plot of the experimental versus predicted values for the validation set is provided in supporting information Section 9.3.

7.9.Comments on the external validation of the model:

The choice of proportions between the training set and the validation set as well as the splitting method helped in accurately evaluating the model and covering most of the training set chemical space. This goal was accomplished without the need to do a structural sampling that usually shows over-optimistic evaluation of the predictivity or a complete random selection that risks biasing the evaluation towards a certain region of the chemical space.

8.Providing a mechanistic interpretation - OECD Principle 5

8.1.Mechanistic basis of the model:

The model descriptors were selected statistically but they can also be mechanistically interpreted.KOC is the ratio between the concentration of a chemical adsorbed by the soil normalized to soil organic carbon and the concentration dissolved in the soil water. Thussoil sorption is closely related to water solubility and logP. Therefore, the chemical features which determine the soil sorption are similar to those related to water solubility and logP.
In particular, size related descriptors since larger compounds tend to have higher soil sorption because they do have lower water solubility.
Also elctronic profile descriptors related to charges and to charge distribution are of high importance: the presence of an active functional group next to carbon leads to better water solubility, likewise higher polarity leads to better water solubility. The selected logP and water solubility related descriptors employed in the KOC model are the following:
CrippenLogP: Estimate of partition coefficients.
nHBAcc: Number of hydrogen bond acceptors (using CDK HBondAcceptorCountDescriptor algorithm)
SpDiam_Dzi: Barysz matrix: Spectral diameter from Barysz matrix / weighted by first ionization potential.
SsssN: Sum of atom-type E-State: >N-
VP-1: Chi path: Valence path, order 1
MPC3: Path counts: Molecular path count of order 3.TPC: Path counts: Total path count (up to order 10). MPC6: Path counts: Molecular path count of order 6.topoDiameter: Topological diameter (maximum atom eccentricity)
AATS1m: Average Broto-Moreau autocorrelation - lag 1 / weighted by mass.VABC: Van der Waals volumeATS1v: Broto-Moreau autocorrelation - lag 1 / weighted by van der Waals volumes.

8.2.A priori or a posteriori mechanistic interpretation:

A posteriori mechanistic interpretation.

8.3.Other information about the mechanistic interpretation:

For more details and full reference, see references in Section 4.3 and Section 9.2.

9.Miscellaneous information

9.1.Comments:

This QSAR model for LogKoc prediction is part of the NCCT_Models Suite that is a free and open-source standalone application for the prediction of physicochemical properties and environmental fate of chemicals. This application is available in the Supporting informationSection 9.3of this report and in the paperref 2 Section 2.7. The detailed results of this suite of models applied on more than 700k DSSTox chemicals are available on the iCSS chemistry dashboard (https://comptox.epa.gov/dashboard).

This current version of the model is mainly based on curated and standardized data collected from the Physprop database. All NCCT_Models are designed to fulfil the requirement of the 5 OECD principles to ensure transparency and reproducibility of the results. In order to predict new chemicals, the models only require 2D chemical structures that are used to calculate molecular descriptors by PaDEL 2.21 software. Then a simple weighted kNN algorithm is used to make the prediction based on the observed values of the k closest molecules. All models showed high robustness and statistics stability between training, 5-fold cross-validation and the external validation set.

Considering the full applicability domain of the 729 chemicals with available data and the same models parameters described earlier, the calibration statistics would be an $R^2$of 0.78 and an RMSE of 0.57.

9.2.Bibliography:

James R. Baker, James R. Mihelcic, Aleksandar Sabljic, Reliable QSAR for estimating Koc for persistent organic pollutants: correlation with molecular connectivity indices, Chemosphere, Volume 45, Issue 2, October 2001, Pages 213-221 http://www.sciencedirect.com/science/article/pii/S0045653500003398

9.3.Supporting information:

10.Summary (JRC QSAR Model Database)

10.1.QMRF number:

Q17-26-0017

10.2.Publication date:

2017-09-21

10.3.Keywords:

Organic carbon-sorption partition coefficient;PaDEL;OPERA;

10.4.Comments:

To be entered by JRC
