# Supplementary figures and images for: OPERA models for predicting physicochemical properties and environmental fate endpoints
Source: J Cheminform. 2018 Mar 8;10:10. doi: 10.1186/s13321-018-0263-1 (PMC5843579; doi:10.1186/s13321-018-0263-1)

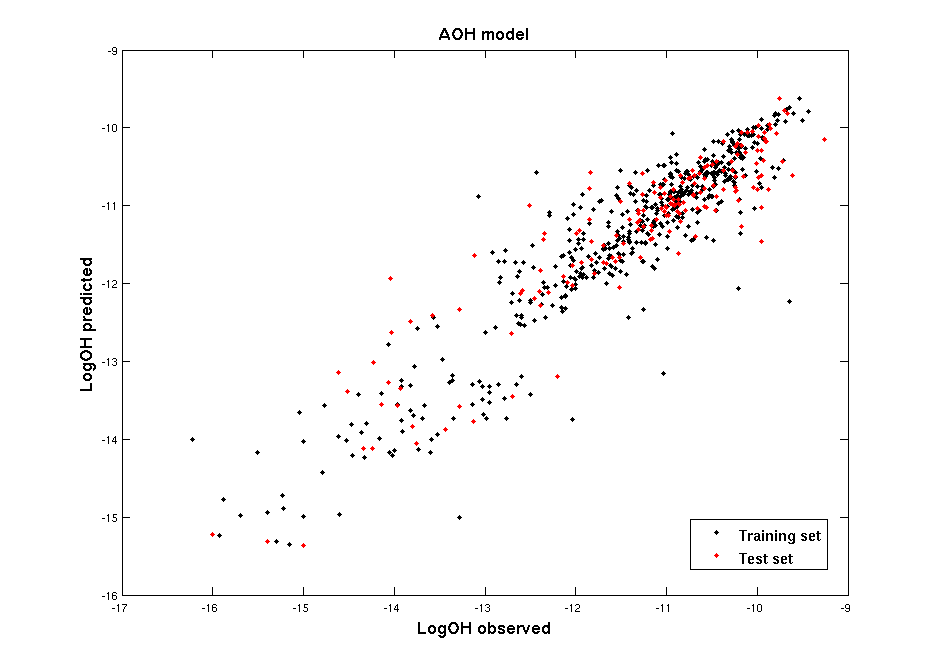

Supplement: Supplementary file 1 — Additional file 1: S1. Training and test sets of the models with the corresponding JRC validated QMRFs. [file 13321_2018_263_MOESM1_ESM.zip › OPERA_AOH/AOH.png]

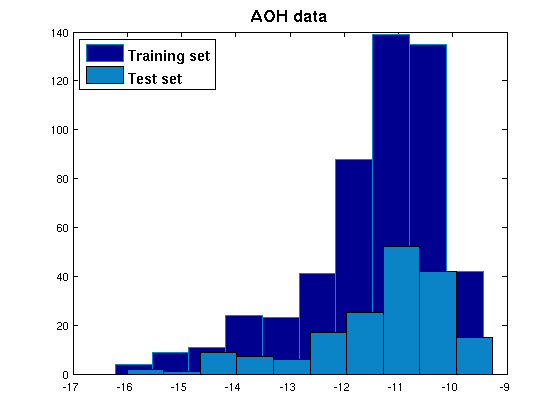

Supplement: Supplementary file 1 — Additional file 1: S1. Training and test sets of the models with the corresponding JRC validated QMRFs. [file 13321_2018_263_MOESM1_ESM.zip › OPERA_AOH/AOH_hist.tif]

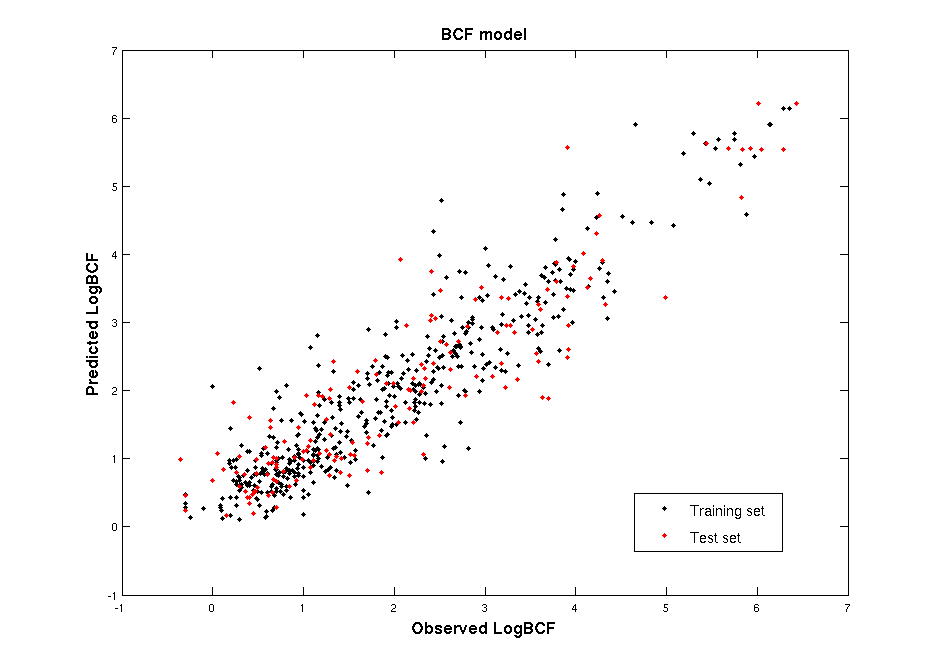

Supplement: Supplementary file 1 — Additional file 1: S1. Training and test sets of the models with the corresponding JRC validated QMRFs. [file 13321_2018_263_MOESM1_ESM.zip › OPERA_BCF/BCF.png]

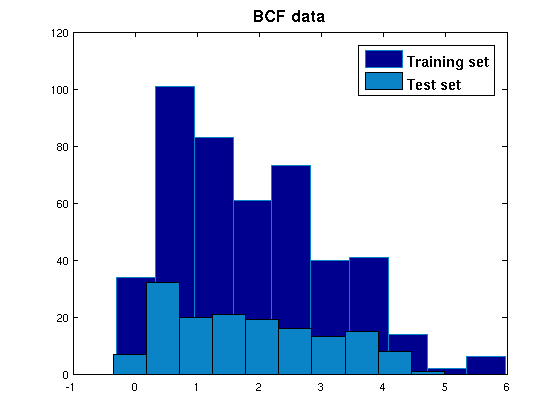

Supplement: Supplementary file 1 — Additional file 1: S1. Training and test sets of the models with the corresponding JRC validated QMRFs. [file 13321_2018_263_MOESM1_ESM.zip › OPERA_BCF/BCF_hist.tif]

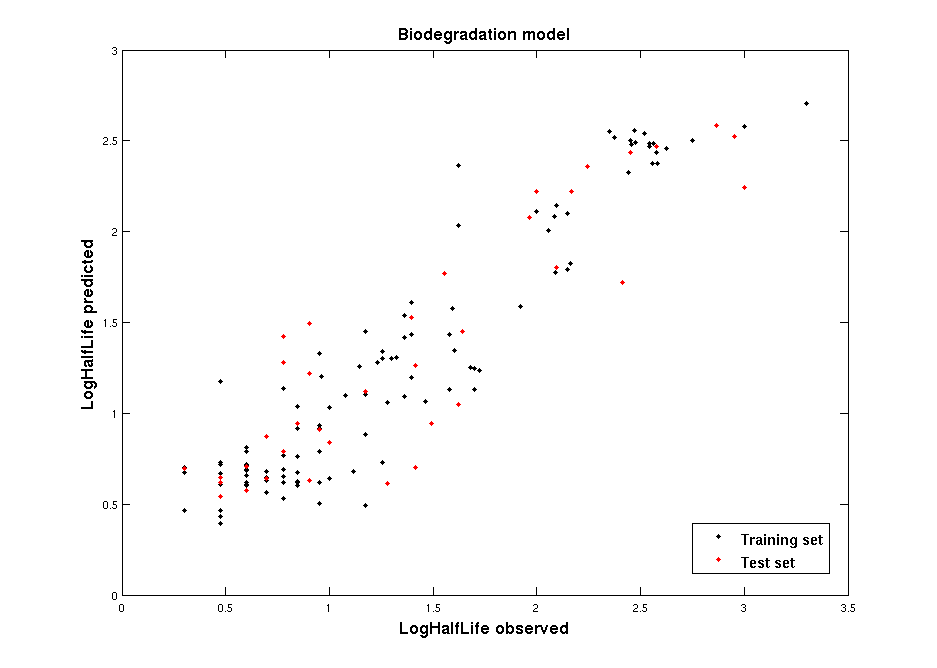

Supplement: Supplementary file 1 — Additional file 1: S1. Training and test sets of the models with the corresponding JRC validated QMRFs. [file 13321_2018_263_MOESM1_ESM.zip › OPERA_BioHL/BioHL.png]

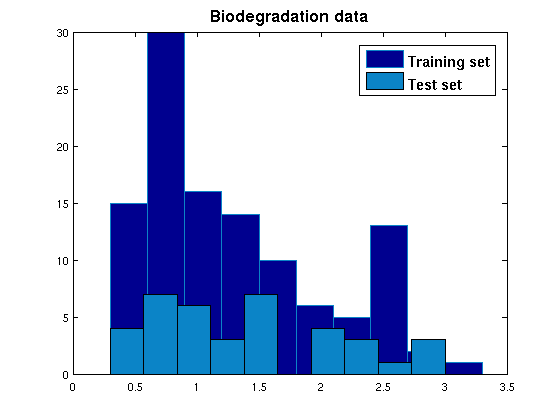

Supplement: Supplementary file 1 — Additional file 1: S1. Training and test sets of the models with the corresponding JRC validated QMRFs. [file 13321_2018_263_MOESM1_ESM.zip › OPERA_BioHL/BioHL_hist.tif]

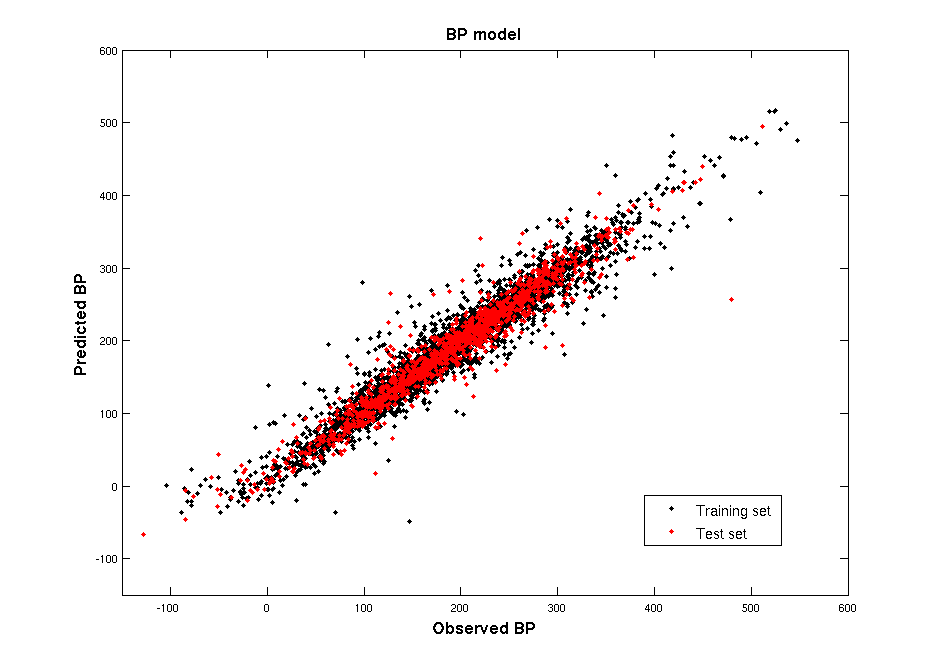

Supplement: Supplementary file 1 — Additional file 1: S1. Training and test sets of the models with the corresponding JRC validated QMRFs. [file 13321_2018_263_MOESM1_ESM.zip › OPERA_BP/BP.png]

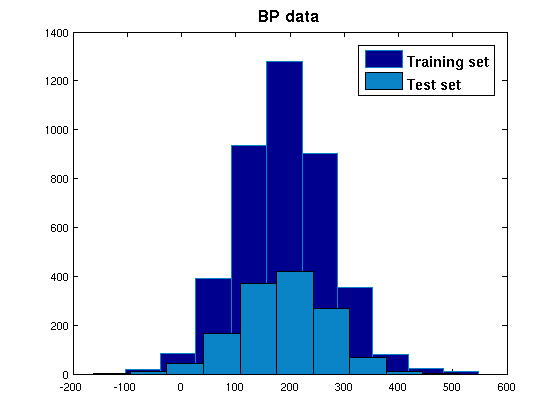

Supplement: Supplementary file 1 — Additional file 1: S1. Training and test sets of the models with the corresponding JRC validated QMRFs. [file 13321_2018_263_MOESM1_ESM.zip › OPERA_BP/BP_hist.tif]

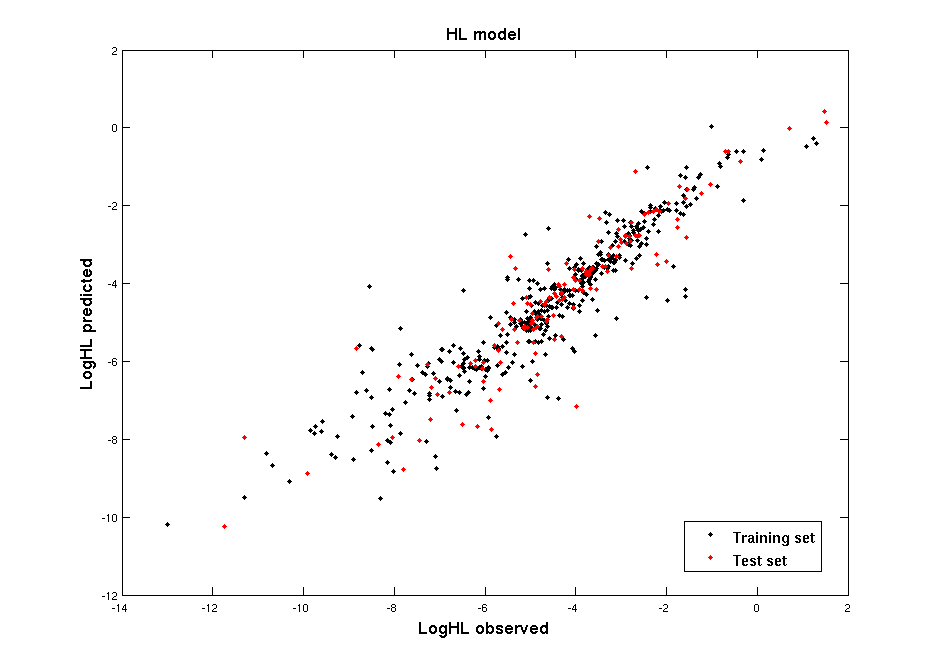

Supplement: Supplementary file 1 — Additional file 1: S1. Training and test sets of the models with the corresponding JRC validated QMRFs. [file 13321_2018_263_MOESM1_ESM.zip › OPERA_HL/HL.png]

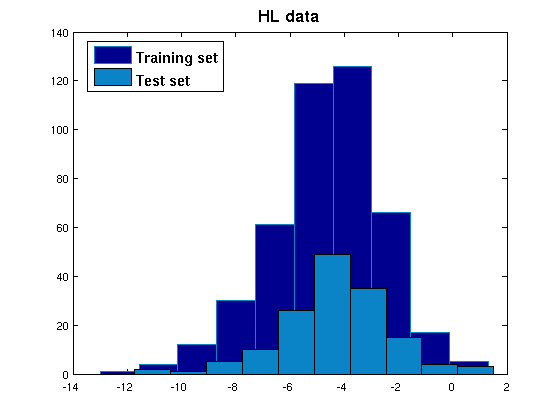

Supplement: Supplementary file 1 — Additional file 1: S1. Training and test sets of the models with the corresponding JRC validated QMRFs. [file 13321_2018_263_MOESM1_ESM.zip › OPERA_HL/HL_hist.tif]

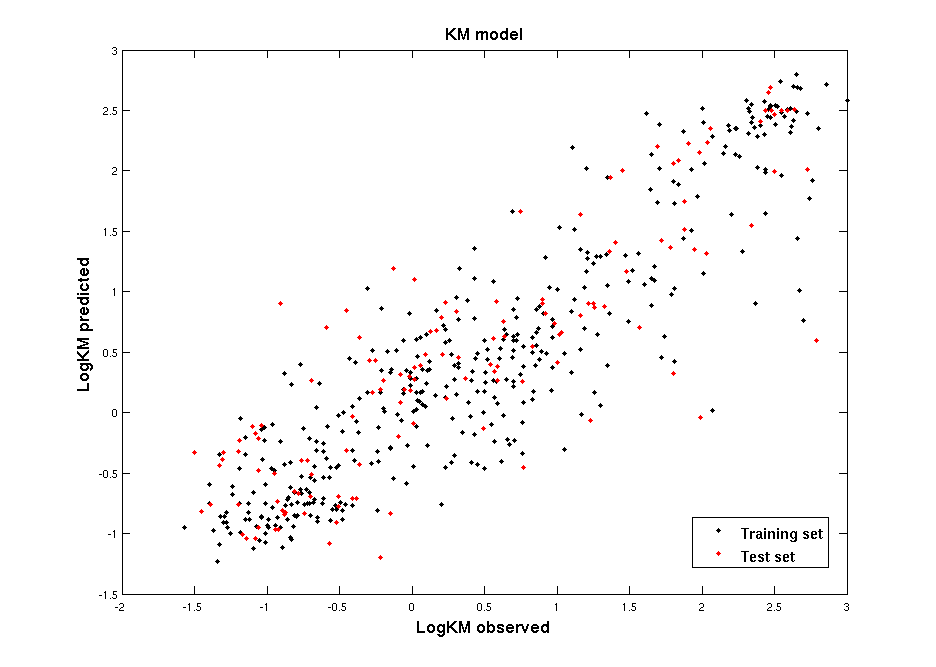

Supplement: Supplementary file 1 — Additional file 1: S1. Training and test sets of the models with the corresponding JRC validated QMRFs. [file 13321_2018_263_MOESM1_ESM.zip › OPERA_KM/KM.png]

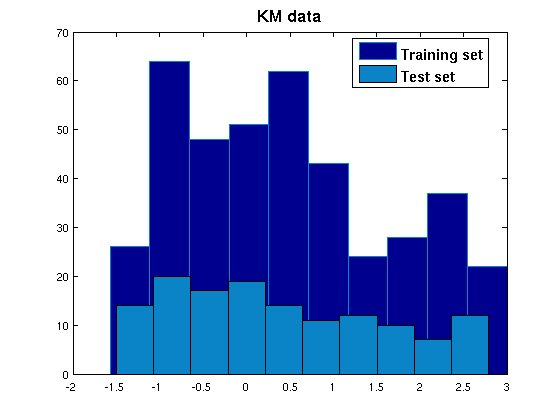

Supplement: Supplementary file 1 — Additional file 1: S1. Training and test sets of the models with the corresponding JRC validated QMRFs. [file 13321_2018_263_MOESM1_ESM.zip › OPERA_KM/KM_hist.tif]

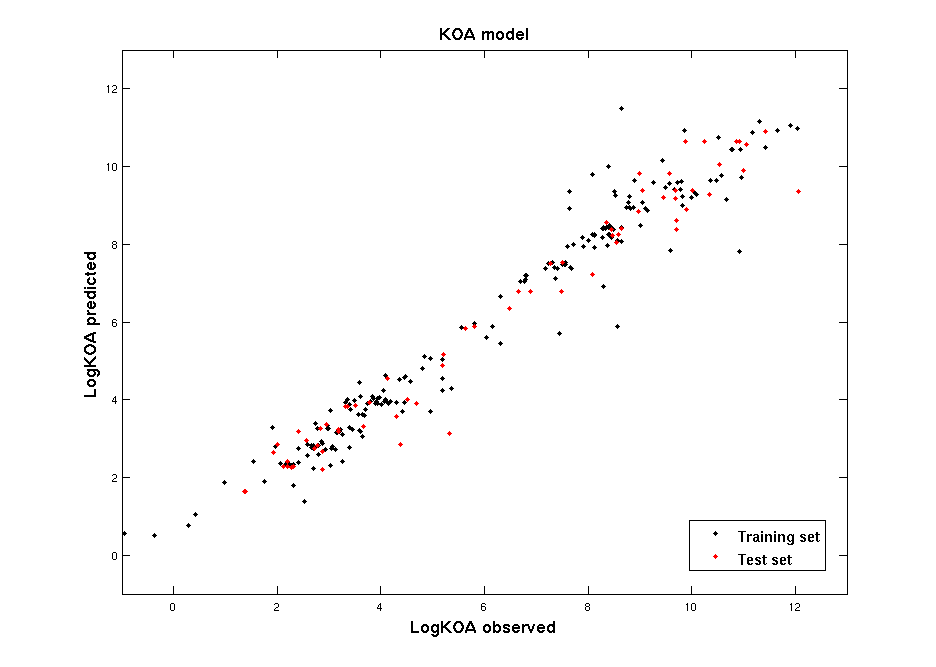

Supplement: Supplementary file 1 — Additional file 1: S1. Training and test sets of the models with the corresponding JRC validated QMRFs. [file 13321_2018_263_MOESM1_ESM.zip › OPERA_KOA/KOA.png]

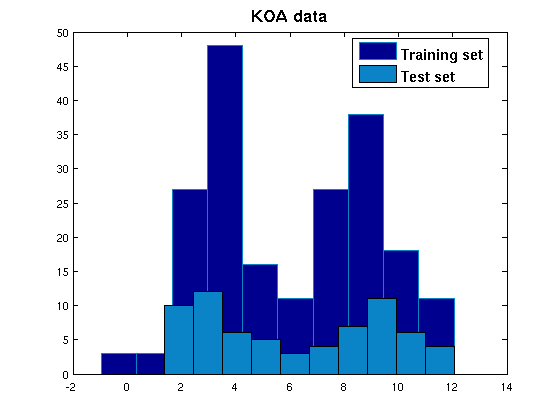

Supplement: Supplementary file 1 — Additional file 1: S1. Training and test sets of the models with the corresponding JRC validated QMRFs. [file 13321_2018_263_MOESM1_ESM.zip › OPERA_KOA/KOA_hist.tif]

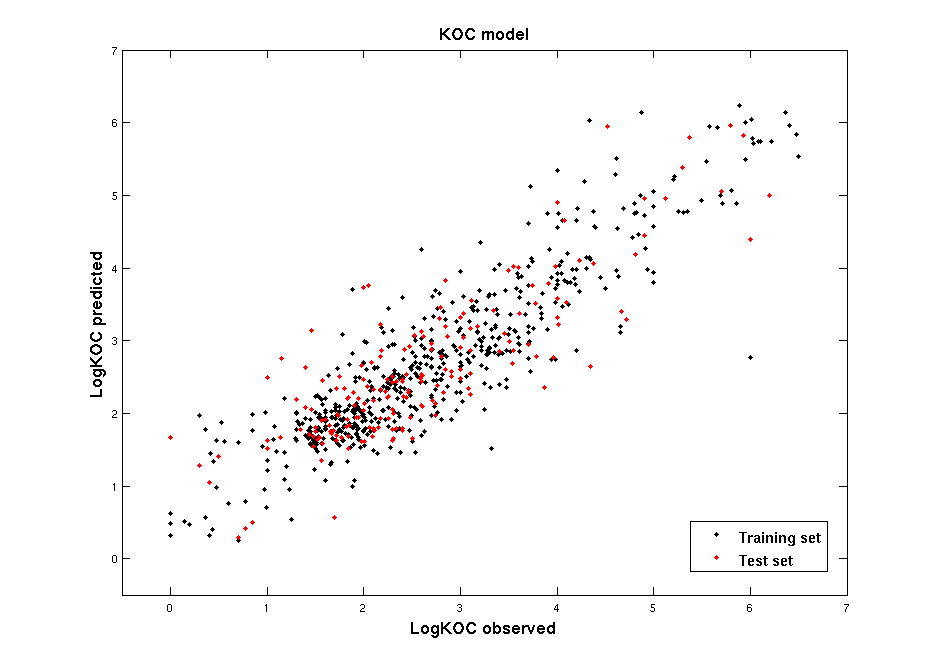

Supplement: Supplementary file 1 — Additional file 1: S1. Training and test sets of the models with the corresponding JRC validated QMRFs. [file 13321_2018_263_MOESM1_ESM.zip › OPERA_KOC/KOC.png]

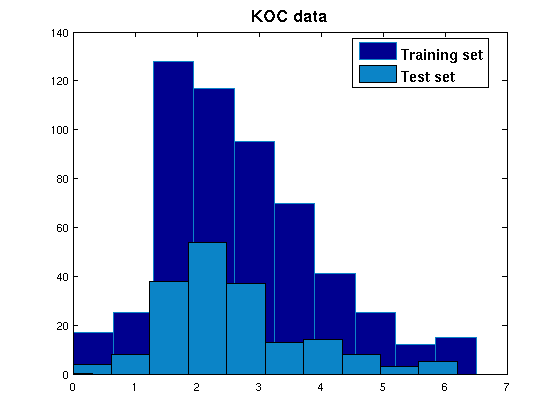

Supplement: Supplementary file 1 — Additional file 1: S1. Training and test sets of the models with the corresponding JRC validated QMRFs. [file 13321_2018_263_MOESM1_ESM.zip › OPERA_KOC/KOC_hist.tif]

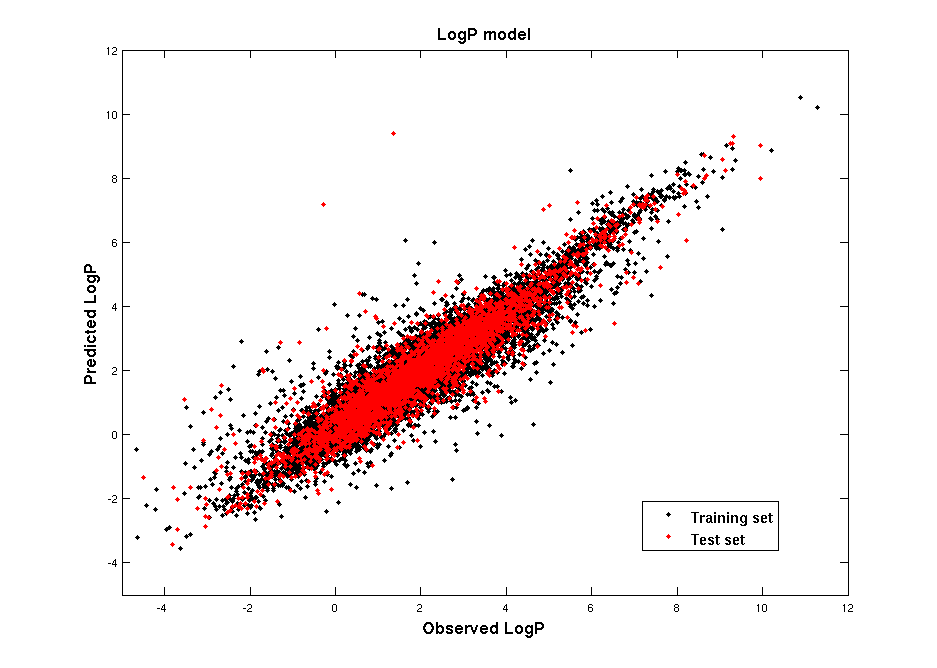

Supplement: Supplementary file 1 — Additional file 1: S1. Training and test sets of the models with the corresponding JRC validated QMRFs. [file 13321_2018_263_MOESM1_ESM.zip › OPERA_LogP/LogP.png]

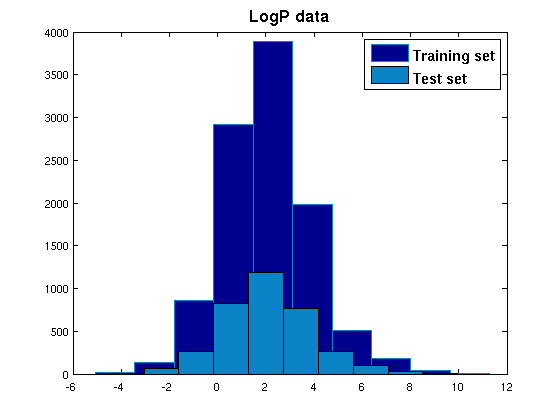

Supplement: Supplementary file 1 — Additional file 1: S1. Training and test sets of the models with the corresponding JRC validated QMRFs. [file 13321_2018_263_MOESM1_ESM.zip › OPERA_LogP/LogP_hist.tif]

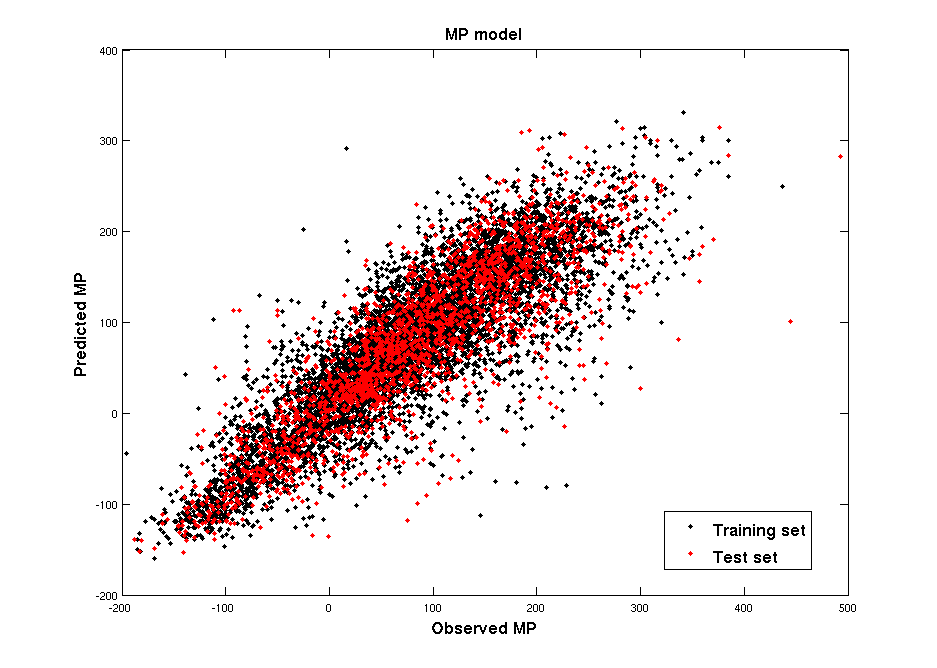

Supplement: Supplementary file 1 — Additional file 1: S1. Training and test sets of the models with the corresponding JRC validated QMRFs. [file 13321_2018_263_MOESM1_ESM.zip › OPERA_MP/MP.png]

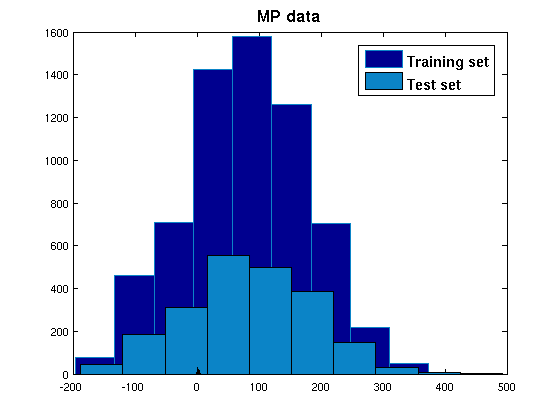

Supplement: Supplementary file 1 — Additional file 1: S1. Training and test sets of the models with the corresponding JRC validated QMRFs. [file 13321_2018_263_MOESM1_ESM.zip › OPERA_MP/MP_hist.tif]

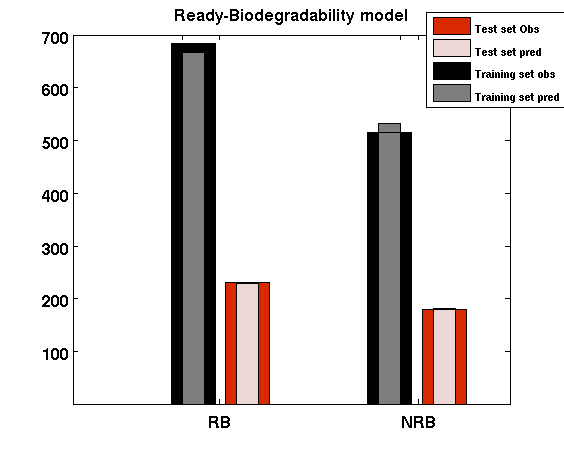

Supplement: Supplementary file 1 — Additional file 1: S1. Training and test sets of the models with the corresponding JRC validated QMRFs. [file 13321_2018_263_MOESM1_ESM.zip › OPERA_RBioDeg/RBiodeg.tif]

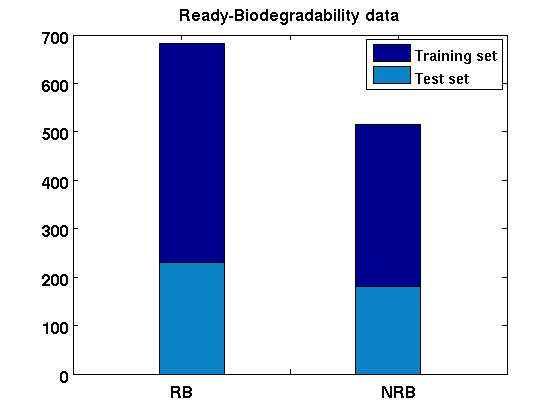

Supplement: Supplementary file 1 — Additional file 1: S1. Training and test sets of the models with the corresponding JRC validated QMRFs. [file 13321_2018_263_MOESM1_ESM.zip › OPERA_RBioDeg/Rbiodeg_hist.png]

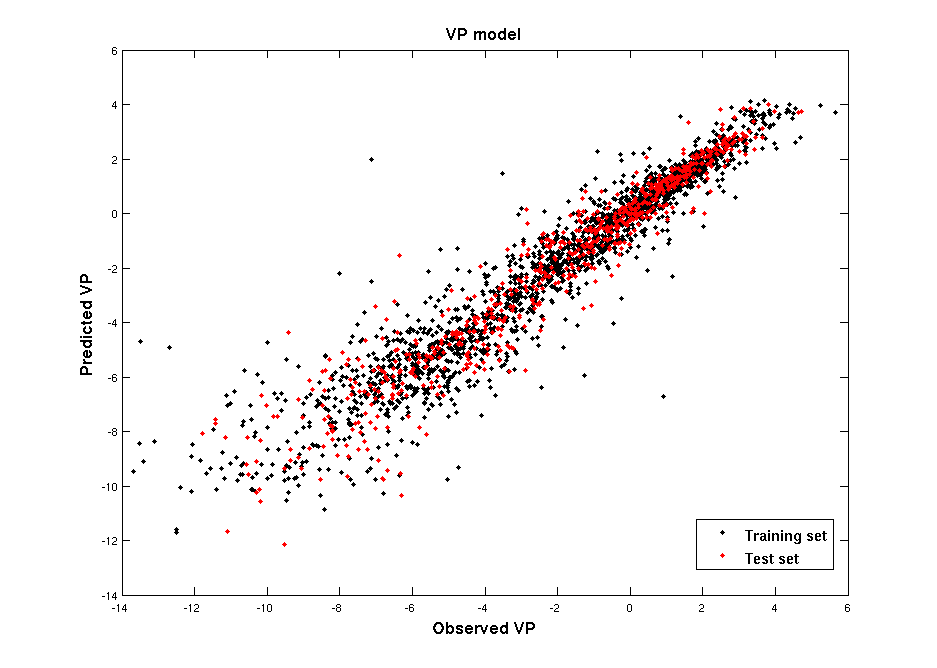

Supplement: Supplementary file 1 — Additional file 1: S1. Training and test sets of the models with the corresponding JRC validated QMRFs. [file 13321_2018_263_MOESM1_ESM.zip › OPERA_VP/VP.png]

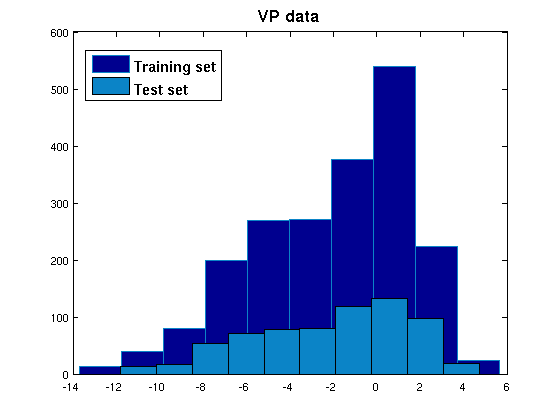

Supplement: Supplementary file 1 — Additional file 1: S1. Training and test sets of the models with the corresponding JRC validated QMRFs. [file 13321_2018_263_MOESM1_ESM.zip › OPERA_VP/VP_hist.tif]

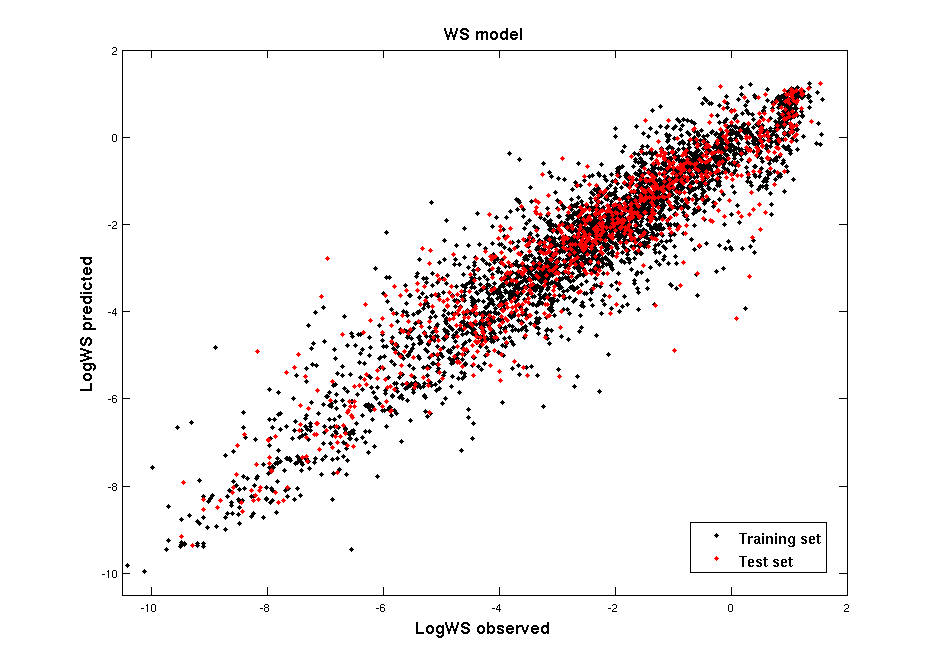

Supplement: Supplementary file 1 — Additional file 1: S1. Training and test sets of the models with the corresponding JRC validated QMRFs. [file 13321_2018_263_MOESM1_ESM.zip › OPERA_WS/WS.png]

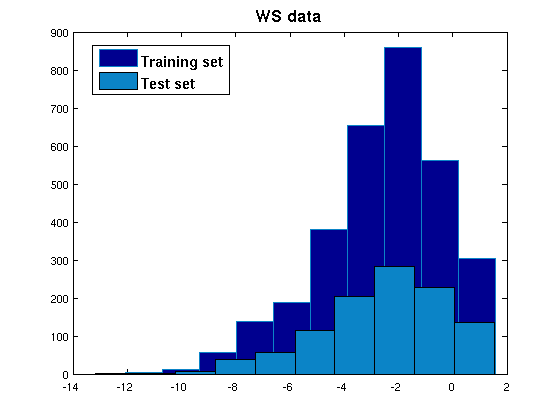

Supplement: Supplementary file 1 — Additional file 1: S1. Training and test sets of the models with the corresponding JRC validated QMRFs. [file 13321_2018_263_MOESM1_ESM.zip › OPERA_WS/WS_hist.tif]
